# Nanomaterial- and shape-dependency of TLR2 and TLR4 mediated signaling following pulmonary exposure to carbonaceous nanomaterials in mice

**DOI:** 10.1186/s12989-021-00432-z

**Published:** 2021-10-30

**Authors:** Pernille Høgh Danielsen, Katja Maria Bendtsen, Kristina Bram Knudsen, Sarah Søs Poulsen, Tobias Stoeger, Ulla Vogel

**Affiliations:** 1 National Research Centre for the Working Environment, Copenhagen, Denmark; 2 Comprehensive Pneumology Center (CPC)/Institute of Lung Biology and Disease (ILBD) Helmholtz Zentrum München, Neuherberg, Germany; 3 DTU Food, Technical University of Denmark, Kgs. Lyngby, Denmark

**Keywords:** Toll-like receptors, Nanomaterials, Inflammation, Acute phase response, Knockout mice

## Abstract

**Background:**

Pulmonary exposure to high doses of engineered carbonaceous nanomaterials (NMs) is known to trigger inflammation in the lungs paralleled by an acute phase response. Toll-like receptors (TLRs), particularly TLR2 and TLR4, have recently been discussed as potential NM-sensors, initiating inflammation. Using *Tlr2* and *Tlr4* knock out (KO) mice, we addressed this hypothesis and compared the pattern of inflammation in lung and acute phase response in lung and liver 24 h after intratracheal instillation of three differently shaped carbonaceous NMs, spherical carbon black (CB), multi-walled carbon nanotubes (CNT), graphene oxide (GO) plates and bacterial lipopolysaccharide (LPS) as positive control.

**Results:**

The LPS control confirmed a distinct TLR4-dependency as well as a pronounced contribution of TLR2 by reducing the levels of pulmonary inflammation to 30 and 60% of levels in wild type (WT) mice. At the doses chosen, all NM caused comparable neutrophil influxes into the lungs of WT mice, and reduced levels were only detected for GO-exposed *Tlr2* KO mice (35%) and for CNT-exposed *Tlr4* KO mice (65%). LPS-induced gene expression was strongly TLR4-dependent. CB-induced gene expression was unaffected by TLR status. Both GO and MWCNT-induced *Saa1* expression was TLR4-dependent. GO-induced expression of *Cxcl2*, *Cxcl5*, *Saa1* and *Saa3* were TLR2-dependent. NM-mediated hepatic acute phase response in terms of liver gene expression of *Saa1* and *Lcn2* was shown to depend on TLR2 for all three NMs. TLR4, in contrast, was only relevant for the acute phase response caused by CNTs, and as expected by LPS.

**Conclusion:**

TLR2 and TLR4 signaling was not involved in the acute inflammatory response caused by CB exposure, but contributed considerably to that of GO and CNTs, respectively. The strong involvement of TLR2 in the hepatic acute phase response caused by pulmonary exposure to all three NMs deserves further investigations.

**Supplementary Information:**

The online version contains supplementary material available at 10.1186/s12989-021-00432-z.

## Background

Carbonaceous nanomaterials (NMs) including carbon black (CB), carbon nanotubes (CNTs) and graphene oxide (GO) are commonly produced and applied in a wide range of industrial and consumer products [1–3]. Potential occupational exposure of workers via inhalation can lead to adverse health effects and cause lung inflammation as well as systemic effects [4].

The interaction of NMs with the innate immune system is important for recognition and elimination, which provides the first line of defense against foreign material in the body [5, 6]. It has been hypothesized that the innate immune system sense NMs directly or via surface-adsorbed biomolecules by pattern recognition receptors (PRRs) on the cell surface. Toll-like receptors (TLRs) belong to the family of PRRs and are activated upon binding with several ligands, often referred to as senses pathogen-associated molecular patterns (PAMPs) and damage-associated molecular patterns (DAMPs) [7] initiating a signaling cascade leading to the release of cytokines and chemokines which recruits immune cells [8]. In the airways, TLR2 and TLR4 are the most relevant TLRs, and these play a critical role in the progression of pulmonary diseases [9, 10]. TLR4 is well known for its recognition of bacterial lipopolysaccharide (LPS) [11], whereas TLR2 recognizes lipoproteins from various pathogens [12]. However, TLR2 and TLR4 also recognize endogenous host-derived DAMP ligands, usually produced or released as result of tissue injury [13]. DAMPs released from cells following tissue injury or cell death can be recognized by macrophages and can thus trigger the inflammatory response by different pathways, including TLR [14]. Different DAMPs interact with different TLR receptors, e.g. heat shock proteins (TLR2, TLR4), HMGB1 (TLR2, TLR4, (also RAGE)) and SAA (TLR2) [13]. Carbonaceous NMs (C60, SWCNT and GO) were found to cause release of HMGB1 from mice lung cells, especially macrophages, leading to activation of the RAGE pathway (TLR pathway was not investigated) [15]. Gold nanoparticles were found to bind HMGB1 in the lysosomes of macrophages, which were associated with inhibition of TLR9 function, other TLRs were not investigated [16]. The coarse and fine fractions of airborne particulate matter caused induction of the heat shock protein 70 in vitro, whereas the ultrafine fraction did not [17]. TLR2 has furthermore been proposed to be a functional receptor for the acute phase protein serum amyloid A (SAA) [18]. Different isoforms of SAA exists; in both mice and humans, SAA1 and SAA2 are inducible by inflammatory cytokines [19], but whereas *Saa3* is considered a pseudogene in humans, it is the most differentially regulated gene in lung tissue of mice exposed to various types of NMs [20–23]. Acute phase response is an established risk factor for cardiovascular disease in a prospective epidemiological study [24]. Acute phase response and the accompanying inflammatory response are strongly associated to increased risk of atherosclerosis [19, 20, 25].

Few studies have investigated the possible direct interaction between NMs and TLRs. Computational studies indicate that carbon nanostructures such as present on C60 or CNTs might bind to the internal hydrophobic pockets of some TLRs, in particular TLR2, and TLR4 [26]. One study suggests that single-walled CNTs trigger chemokine production through a direct interaction with TLR4 [27], another study using gold nanoparticles suggests that the inflammatory response in lung epithelial cells was induced following SAA1-TLR2 ligand-receptor interaction [28].

Previous studies show that pulmonary exposure to poorly soluble carbon-based nanoparticles, such as CB, induce a dose-dependent neutrophil influx and pulmonary acute phase response at day 1 post-exposure that declines over time [29–31]. In contrast, pulmonary exposure to differently shaped CNTs and GO induce a stronger dose-dependent neutrophil influx and pulmonary acute phase response that continues to increase until day 3, and the pulmonary response is accompanied by a hepatic acute phase response 1 day post-exposure [32–36]. It has been speculated that the bending structure of GO may trigger TLR activation or that GO, with a high level of hydroxylation, may trigger similar responses as LPS [35]. The observed differences in response may be due to differences in the interaction with cells and the subsequent initiation of the inflammatory response following exposure to NMs, however, the initiating mechanisms are largely unknown.

In the present study, we hypothesize that inflammatory and acute phase responses can be mediated by different signaling pathways, and we furthermore hypothesize that CNT and GO may initiate a physicochemical-dependent inflammatory response which is mediated, at least partially, by the same TLR-dependent signaling pathway as LPS, whereas CB-induced inflammation presumably is not. In order to investigate this, we performed (1) two pilot studies in mice using the TLR-antagonist Sparstolonin B (methods and results are found in Additional file 1) and subsequently (2) the main study where we exposed *Tlr4* and *Tlr2* knockout (KO) mice to CB, CNT, GO and LPS by intratracheal (i.t) instillation and compared the response to wild type (WT) mice. The influx of inflammatory cells in the lungs, expression of inflammation markers and acute phase response in lung and liver were assessed 1 day post-exposure.

## Results

### Characterization of the NMs

The key physico-chemical characteristics of the NMs are summarized in Table 1. Dynamic light scattering was used to determine the hydrodynamic number-based size distributions of NMs in the instillation suspensions (3.24 mg/ml in Nanopure Diamond water with 2% mouse serum). The hydrodynamic number-based size distribution showed unimodal peaks for CB and CNT at 44 nm and 342 nm, respectively and was slightly bimodal for GO showing a small peak at 28 nm and a larger around 73 nm (Additional file 1: Fig. S1). The intensity-based size distributions are shown in Additional file 1: Fig. S2. The z-average size and polydispersity index are shown in Table S1 (Additional file 1).

**Table 1:** Key characteristics of the NMs

Name	NM-type	Source	Length (µm)	Diameter (nm)	Shape	BET (m^2/^g)	Purity (%)	Carbon (wt%)	Carbon (mmol/g)	Oxygen (mmol/g)	Endotoxin (EU/mg)	Reference
CB	Carbon Black-Printex 90	Degussa-Hüls, Germany	–	14	Sphere	314	> 99	99.3	71.77	1.45	0.14	Bengtson et al. [63]
CNT	MWCNT-Mitsui7	Hadoga Chemical industry, Japan	5.73	74	Tube	26	> 99	99.6	–	0.08	0.51	Jackson et al. [65]
GO	Graphene oxide	Graphena, Spain	2–3^a^	^b^	Plate	–	> 98	43.3	36.05	26.56	1.77	Bengtson et al. [63]

^a^Lateral size

^b^2–3 stacked layers, thickness not determined

The dose levels of CB (162 µg), CNT (54 µg) and GO (18 µg) were selected based on previous studies [35, 37] to induce comparable levels of inflammation but not overt toxicity in terms of alveolar injury and systemic toxicity e.g. weight loss. Protein content in BAL fluid was used as biomarker of lung toxicity and alveolar barrier integrity. The protein content was unaffected by exposure and mouse *Tlr* status, indicating that the exposures did not induce overt toxicity (Additional file 1: Fig. S3).

### Cell composition in BAL fluid

**Fig. 1:**
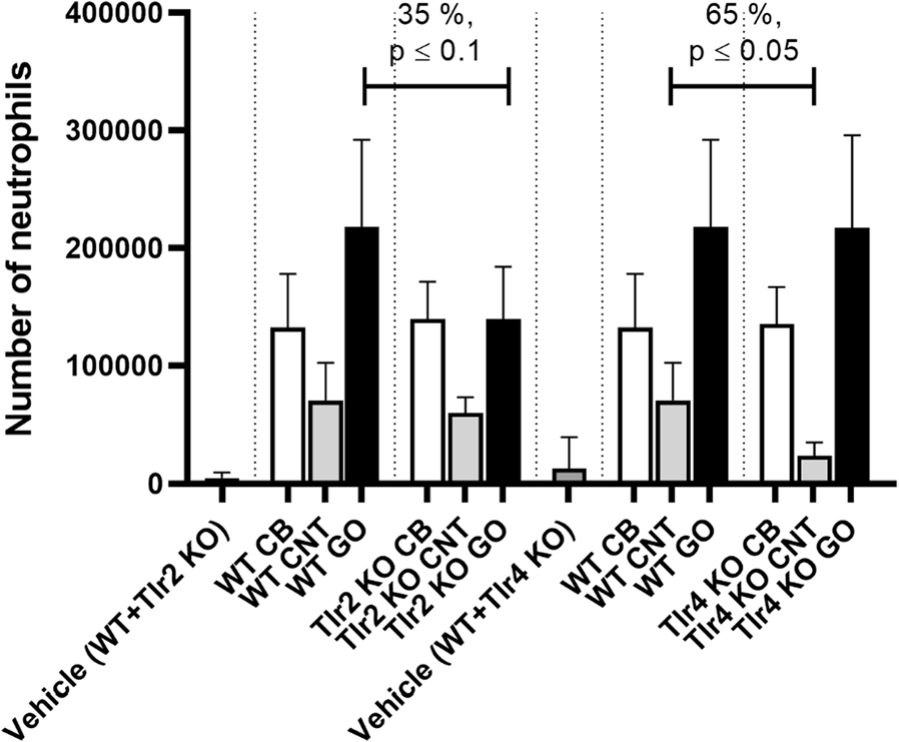
Number of neutrophils in BAL fluid in *Tlr2* KO and *Tlr4* KO mice versus WT mice. All values are presented as mean ± SD. Mann–Whitney test was used to test the differences between WT and KO mice

Influx of inflammatory cells; total number of cells and number of macrophages, lymphocytes, neutrophils, eosinophils and epithelial, in BAL fluid was assessed to monitor the cellular dynamics of the pulmonary inflammatory response. All data are presented in Tables 2 and 3. As expected, the number of total BAL cells, mainly driven by the influx of neutrophils, were significantly increased in WT and *Tlr2* KO mice, but not *Tl4* KO mice after exposure to LPS when compared to the vehicle control. In contrast, all NMs provoked BAL neutrophil levels exceeding control conditions, but the range of increases depended on *Tlr* genotypes. Differences in neutrophil numbers were observed between WT mice and the KO mice, where CNT-exposed *Tlr4* KO mice showed a significant decrease of 65% and GO-exposed *Tlr2* KO mice a decrease of 35% (Fig. 1). Interestingly, BAL eosinophil numbers were increased only upon CB-exposure in WT mice (Tables 2 and 3). Additionally, BAL macrophage numbers decreased to levels below 50% of control for CB-exposed WT mice (Tables 2 and 3).

**Table 2:** BAL cell counts in mice 24 h post exposure

	Control	LPS	CB	CNT	GO
	0 µg	4 µg	162 µg	54 µg	18 µg
WT					
Total BAL cells (× 10^3^)	73.1 ± 34.9	824 ± 404***	191 ± 61.2**	151 ± 33.6**	293 ± 85.9***
Macrophages (× 10^3^)	59.9 ± 19.3	76.8 ± 47.3	23.9 ± 15.4**	50.8 ± 8.74	52.4 ± 13.9
Lymphocytes (× 10^3^)	0.51 ± 0.45	6.14 ± 6.96	1.30 ± 1.35	2.20 ± 1.51	1.61 ± 1.10
Neutrophils (× 10^3^)	4.75 ± 4.93	724 ± 338**	133 ± 45.4***	70.8 ± 31.9**	218 ± 73.9**
Eosinophils (× 10^3^)	8.30 ± 22.8	1.37 ± 3.06	28.2 ± 23.4**	21.3 ± 15.0*	17.1 ± 9.82*
Epithelial (× 10^3^)	4.40 ± 3.60	15.6 ± 14.7*	5.52 ± 3.95	5.61 ± 1.94	4.13 ± 2.65
*Tlr2* KO					
Total BAL cells (× 10^3^)	73.1 ± 34.9	475 ± 106***	184 ± 31.0**	133 ± 47.7*	239 ± 33.2**
Macrophages (× 10^3^)	59.9 ± 19.3	30.1 ± 17.9*	27.1 ± 17.3*	35.9 ± 18.6	81.4 ± 22.9
Lymphocytes (× 10^3^)	0.51 ± 0.45	1.27 ± 1.47	4.19 ± 2.32**^, a^	2.65 ± 2.93*	6.46 ± 7.70
Neutrophils (× 10^3^)	4.75 ± 4.93	405 ± 66.0**	140 ± 31.9**	60.0 ± 13.5**	140 ± 44.1**
Eosinophils (× 10^3^)	8.30 ± 22.8	0.00 ± 0.00	9.65 ± 7.75*	27.0 ± 37.5*	8.45 ± 10.8*
Epithelial (× 10^3^)	4.40 ± 3.60	3.29 ± 3.23	3.13 ± 1.22	7.34 ± 3.93	6.05 ± 1.27

All values are presented as mean ± SD. There are no statistical significant difference between the vehicle controls, therefore these are pooled in the statistical analyses (WT + *Tlr2* KO). A symbol (*) denotes P ≤ 0.05, (**) P ≤ 0.01, (***) P ≤ 0.001 compared to vehicle control. Comparing *Tlr2* KO and WT, a letter (^a^) denotes P ≤ 0.05, (^b^) P ≤ 0.01

**Table 3:** BAL cell counts in mice 24 h post exposure

	Control	LPS	CB	CNT	GO
	0 µg	4 µg	162 µg	54 µg	18 µg
WT					
Total BAL cells (× 10^3^)	89.6 ± 56.3	824 ± 404***	191 ± 61.2**	151 ± 33.6*	293 ± 85.9**
Macrophages (× 10^3^)	67.2 ± 22.6	76.8 ± 47.3	23.9 ± 15.4***	50.8 ± 8.74	52.4 ± 13.9
Lymphocytes (× 10^3^)	0.34 ± 0.31	6.14 ± 6.96	1.30 ± 1.35	2.20 ± 1.51*	1.61 ± 1.10*
Neutrophils (× 10^3^)	12.8 ± 26.9	724 ± 338**	133 ± 45.4***	70.8 ± 31.9*	218 ± 73.9**
Eosinophils (× 10^3^)	10.2 ± 22.4	1.37 ± 3.06	28.2 ± 23.4*	21.3 ± 15.0	17.1 ± 9.82
Epithelial (× 10^3^)	5.98 ± 3.86	15.6 ± 14.7	5.52 ± 3.95	5.61 ± 1.94	4.13 ± 2.65
*Tlr4* KO					
Total BAL cells (× 10^3^)	89.6 ± 56.3	295 ± 423	178 ± 43.1*	53.2 ± 33.4^b^	293 ± 82.0**
Macrophages (× 10^3^)	67.2 ± 22.6	66.8 ± 28.4	14.8 ± 7.30**	21.2 ± 19.5**^, a^	50.7 ± 13.8
Lymphocytes (× 10^3^)	0.34 ± 0.31	0.21 ± 0.42	1.50 ± 0.92*	0.70 ± 0.61	0.82 ± 0.79
Neutrophils (× 10^3^)	12.8 ± 26.9	219 ± 411	135 ± 31.5**	23.9 ± 11.2*^, a^	217 ± 78.9**
Eosinophils (× 10^3^)	10.2 ± 22.4	5.80 ± 8.67	21.4 ± 15.9*	5.03 ± 2.84	19.4 ± 14.8
Epithelial (× 10^3^)	5.98 ± 3.86	3.50 ± 4.05	4.63 ± 2.47	2.36 ± 1.70	5.48 ± 6.31

All values are presented as mean ± SD. There were no statistical significant difference between the vehicle controls, therefore these were pooled in the statistical analyses (WT + *Tlr4* KO). A symbol (*) denotes P ≤ 0.05, (**) P ≤ 0.01, (***) P ≤ 0.001 compared to vehicle control. Comparing *Tlr4* KO and WT, a letter (^a^) denotes P ≤ 0.05, (^b^) P ≤ 0.01

### Inflammatory markers

Pulmonary inflammation was also assessed by quantifying *Il-6, Tnf*, *Cxcl2* and *Cxcl5* mRNA expression levels in lung tissue.

Different types of NMs upregulate the pro-inflammatory genes *Il-6* and *Tnf* in various studies [38–42] and these are among the important cytokines of the acute phase response [43].

**Fig. 2:**
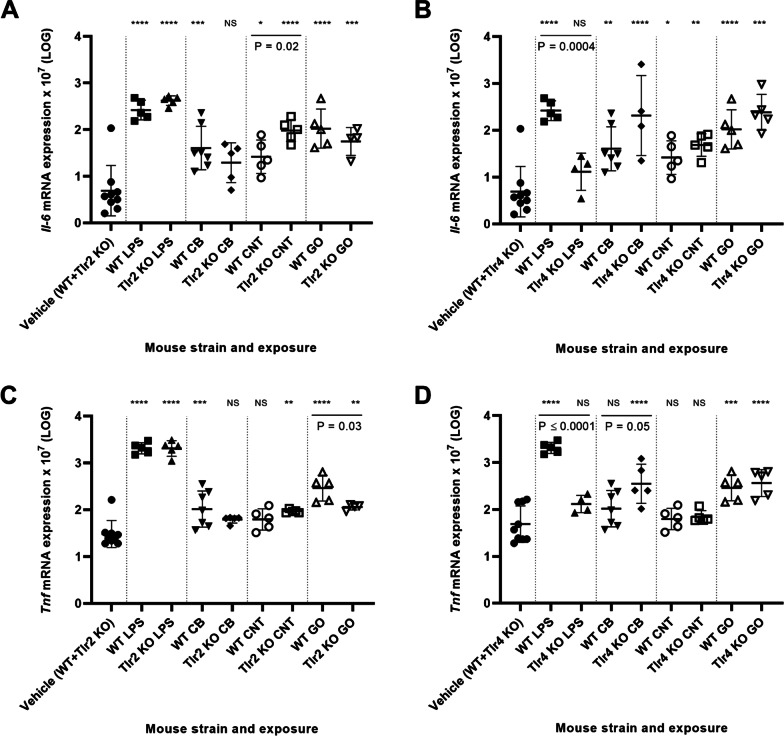
Pulmonary mRNA expression levels of *Il-6* in *Tlr2* KO mice versus WT mice (**A**) and *Tlr4* KO mice versus WT mice (**B**). Pulmonary mRNA expression levels of *Tnf* in *Tlr2* KO mice versus WT mice (**C**) and *Tlr4* KO mice versus WT mice (**D**). All values are log transformed and presented as mean ± SD. A marker (*) denotes P ≤ 0.05, (**) P ≤ 0.01, (***) P ≤ 0.001, (****) P ≤ 0.0001 compared to vehicle control (Dunn’s multiple comparison method). The vertical lines mark statistically significant differences between KO mice and WT mice (unpaired t-test)

As observed on the level of BAL cells, LPS caused a strong pro-inflammatory signature for all genes in WT and *Tlr2* KO mice, but not *Tlr4* KO mice. Overall, NM-exposure increased *Il-6* and *Tnf* mRNA expression levels (Fig. 2A–D, respectively), though predominantly for *Il-6*, and the *Il-6* and *Tnf* mRNA levels correlate closely across exposures and *Tlr* status (r = 0.8370, P ≤ 0.0001) (Additional file 1: Fig. S4). The pro-inflammatory response following NM exposure in terms of *Il-6* and *Tnf* expression was different from the LPS response. Whereas LPS-mediated expression depended on TLR4 (Fig. 2B, 2D) and was independent of TLR2 (Fig. 2A, C), NM-mediated *Il-6* and *Tnf* were independent of TLR4 (Fig. 2B, C), and GO-mediated *Tnf* expression was TLR2 dependent (Fig. 2C)*.* Furthermore, TLR4 deletion increased CB-mediated *Tnf* expression (Fig. 2D) and TLR2 deletion increased CNT-mediated *Il-6* expression (Fig. 2A).

**Fig. 3:**
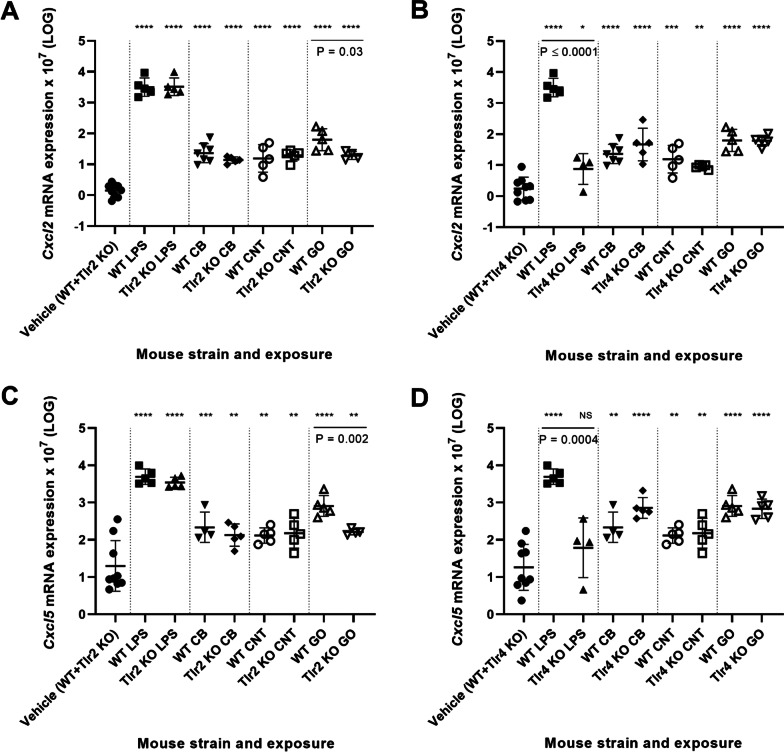
Pulmonary mRNA expression levels of *Cxcl2* in *Tlr2* KO mice versus WT mice (**A**) and *Tlr4* KO mice versus WT mice (**B**). Pulmonary mRNA expression levels of *Cxcl5* in *Tlr2* KO mice versus WT mice (**C**) and *Tlr4* KO mice versus WT mice (**D**). All values are log transformed and presented as mean ± SD. A marker (*) denotes P ≤ 0.05, (**) P ≤ 0.01, (***) P ≤ 0.001, (****) P ≤ 0.0001 compared to vehicle control (Dunn’s multiple comparison method). The vertical lines mark statistically significant differences between KO mice and WT mice (unpaired t-test)

Cxcl2 (MIP-2 (macrophage inflammatory protein 2)) and Cxcl5 are both important chemokines for neutrophil recruitment [44]. Increased *Cxcl5* mRNA levels have been observed in mouse lung after exposure to CB and titanium dioxide [21, 29]. Cxcl5 can under sterile conditions, such as upon CB exposure, be related to epithelial inflammation [45], while Cxcl2 is mainly expressed by leukocytes such as macrophages. NM and LPS exposure significantly increased the *Cxcl2* and *Cxcl5* mRNA levels for all mouse strains, however a significant decrease was observed for GO-induced levels in *Tlr2* KO mice compared to WT mice (Fig. 3A, C). Thus, *Cxcl2* and *Cxcl5* followed the same expression pattern as *Tnf* and *Il-6* and showed a high correlation across exposures and mouse strains (r = 0.8730, P =  < 0.0001) (Additional file 1: Fig. S5). Additionally, *Cxcl2* and *Cxcl5* plotted as function of *Tnf* and *Il-6* showed high correlations (Additional file 1: Fig. S6).

### Pulmonary acute phase response

**Fig. 4:**
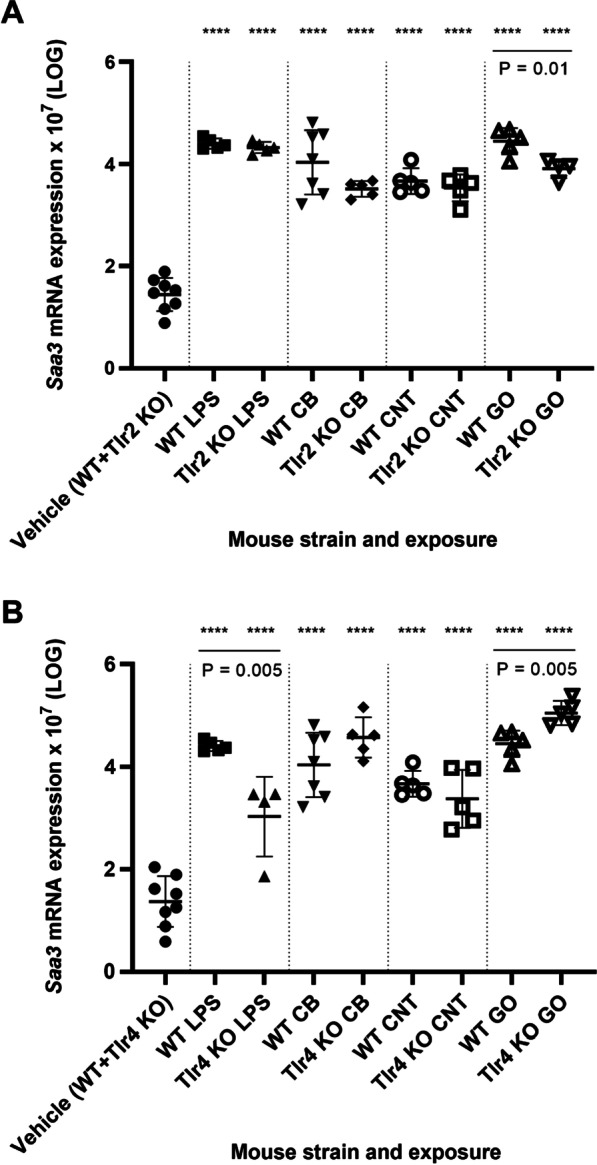
Pulmonary *Saa3* mRNA expression levels in *Tlr2* KO mice versus WT mice (**A**) and *Tlr4* KO mice versus WT mice (**B**). All values are log transformed and presented as mean ± SD. A marker (*) denotes P ≤ 0.05, (**) P ≤ 0.01, (***) P ≤ 0.001, (****) P ≤ 0.0001 compared to vehicle control (Dunn’s multiple comparison method). The vertical lines mark statistically significant differences between KO mice and WT mice (unpaired t-test)

Pulmonary acute phase response was assessed by *Saa3* mRNA expression levels in lung tissue [4, 20, 25]. *Saa3* gene expression was significantly increased for all exposure groups and mouse strains compared to the vehicle control (P < 0.0001) (Fig. 4). Again, when exposed to LPS, the level of *Saa3* mRNA was significantly decreased for the *Tlr4* KO mice compared to WT mice (P < 0.01) (Fig. 4B). Interestingly, upon exposure to GO, *Saa3* mRNA levels were significantly decreased for *Tlr2* KO mice, but increased for *Tlr4* KO mice as compared to WT mice (both P < 0.01) (Fig. 4).

*Saa3* expression levels correlated strongly (r = 0.7909, P < 0.0001) with neutrophil influx across exposures and mouse strains (Additional file 1: Fig. S7A) as previously reported for different NMs where pulmonary acute phase response is induced in parallel with the pulmonary inflammatory response [29, 32, 33, 46–50]. Correlations between *Tnf* and *Saa3*, and *Il-6* and *Saa3* were similarly highly significant (r = 0.8321, P < 0.0001 and r = 0.7962, P < 0.0001) (Additional file 1: Fig. S8). Furthermore, *Lcn2* and *Saa1* mRNA levels in lung tissue, characterized the pulmonary acute phase response, and showed similarly strong correlation with neutrophil influx (Additional file 1: Fig. S7B, S7C). The expression levels of *Lcn2* and *Saa1* were generally much lower in the lung than in liver. However, lung *Saa1* and *Lcn2* expression levels were unaffected by TLR2 status (Figs. 6C, 8C), whereas CNT- and GO-induced *Saa1* and *Lcn2* expression showed a TLR4-dependent pattern (Figs. 6D and 8D).

**Fig. 5:**
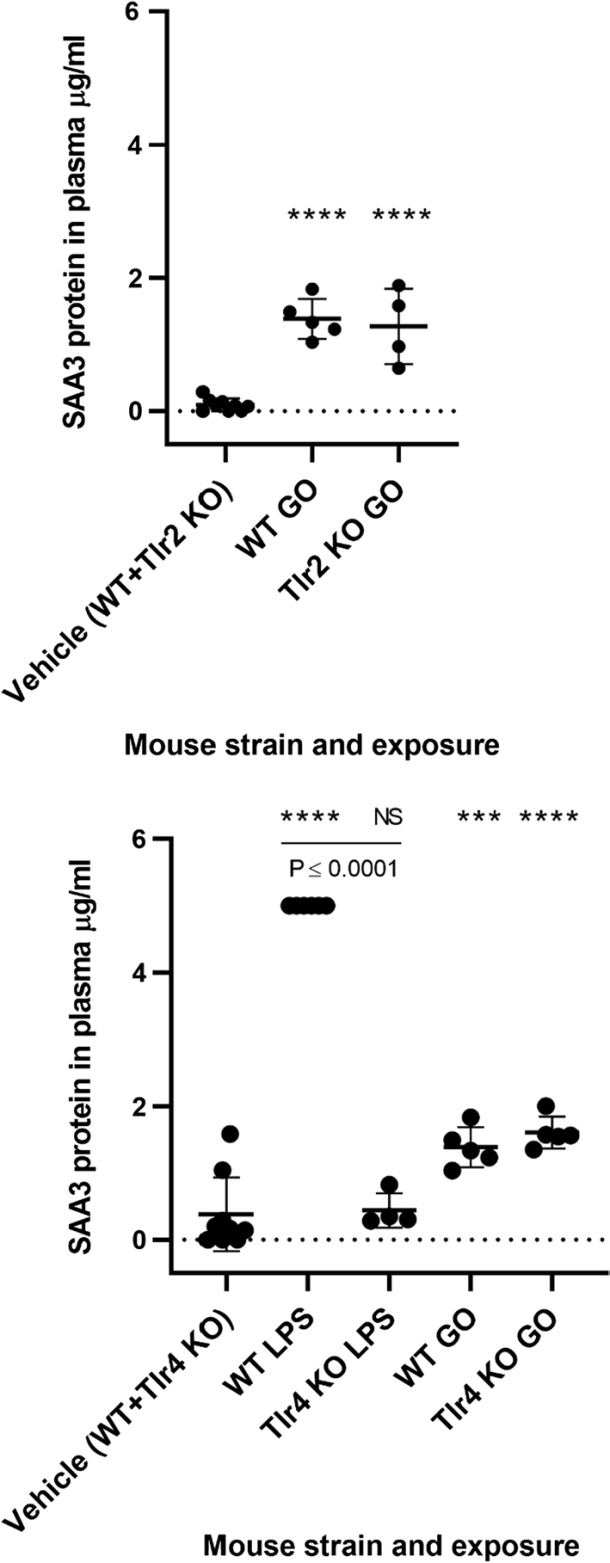
The SAA3 protein levels in plasma. A marker (***) denotes P ≤ 0.001, (****) P ≤ 0.0001 compared to vehicle control (Dunn’s multiple comparison method). The vertical lines mark statistically significant differences between KO mice and WT mice (unpaired t-test)

Pulmonary *Saa3* expression has been shown to be paralleled by increased plasma levels of SAA3 protein in mice exposed to CNT, CB and GO [29, 34, 35], thereby propagating the pulmonary response to a systemic level. In the present study, increased systemic SAA3 protein levels in plasma were confirmed following exposure to LPS and GO (Fig. 5). As expected, LPS-induced plasma levels of SAA3 were significantly lowered in *Tlr4* KO mice, whereas GO-induced SAA3 protein levels were unaffected by TLR status, which is in contrast to the pulmonary response.

### Hepatic acute phase response

Hepatic acute phase response was assessed by the measurement of *Saa1* [32, 34, 51, 52] and *Lcn2* mRNA expression levels [29, 31]. The liver is the major site for SAA1 production induced by inflammation, trauma or stress [53]. The acute phase protein LCN2, which is the mouse homolog of human neutrophil gelatinase associated lipocalin (NGAL) has been shown to be expressed in various tissues promoted by TLR4 and inflammatory cytokines (reviewed in [54]). *Lcn2* is one of the most differentially expressed genes in mouse liver 1 day after instillation with CB [29].

**Fig. 6:**
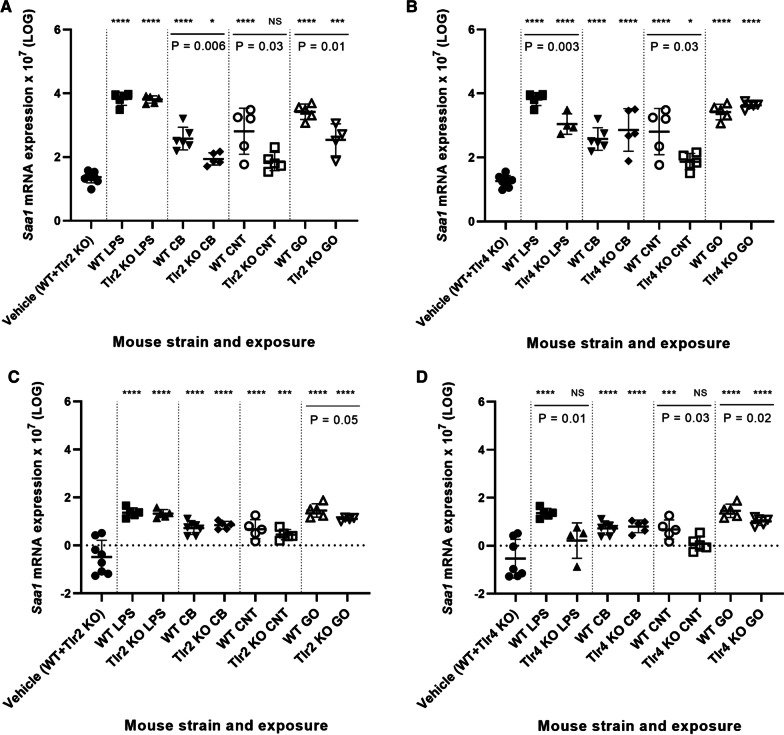
Hepatic mRNA expression levels of *Saa1* in *Tlr2* KO mice versus WT mice (**A**) and *Tlr4* KO mice versus WT mice (**B**). Pulmonary mRNA expression levels of *Saa1* in *Tlr2* KO mice versus WT mice (**C**) and *Tlr4* KO mice versus WT mice (**D**). All values are log transformed and presented as mean ± SD. A marker (*) denotes P ≤ 0.05, (**) P ≤ 0.01, (***) P ≤ 0.001, (****) P ≤ 0.0001 compared to vehicle control (Dunn’s multiple comparison method). The vertical lines mark statistically significant differences between KO mice and WT mice (unpaired t-test)

Overall, *Saa1* gene expression in the liver (Fig. 6A, B) was significantly increased for most NMs and mouse strains compared to the vehicle control. As expected, when exposed to LPS, the level of *Saa1* mRNA was significantly decreased for the *Tlr4* KO mice compared to WT mice (P < 0.01), whereas LPS-induced hepatic *Saa1* expression was again unaffected by TLR2 status. Furthermore, *Saa1* mRNA levels were statistically significantly lowered in liver of mice exposed to CB (P < 0.01, *Tlr2* KO mice), CNT (P < 0.05, both *Tlr2* and *Tlr4* KO mice) and GO (P < 0.01, *Tlr2* KO mice) compared to WT mice.

**Fig. 7:**
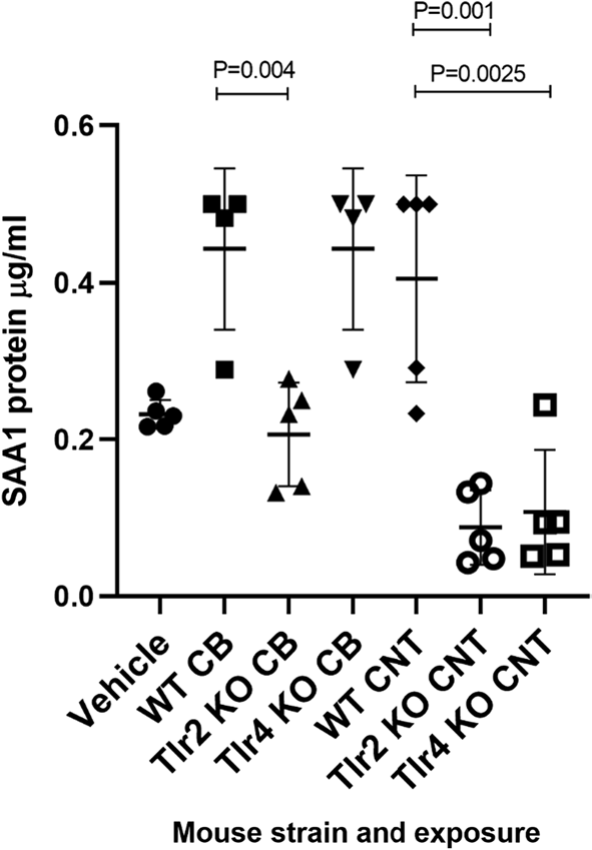
The SAA1/SAA2 protein levels in plasma from CB and CNT-exposed KO mice versus WT mice

The lowered hepatic *Saa1* mRNA levels were reflected in the SAA1/2 protein levels from CB-exposed *Tlr2* KO mice (P = 0.004) and CNT-exposed *Tlr2* and *Tlr4* KO mice (P = 0.001 and P = 0.0025, respectively) (Fig. 7). This match was not observed for *Lcn2* mRNA levels in liver, as only *Tlr4* KO, but not *Tlr2* KO mice showed a CNT exposure related response.

**Fig. 8:**
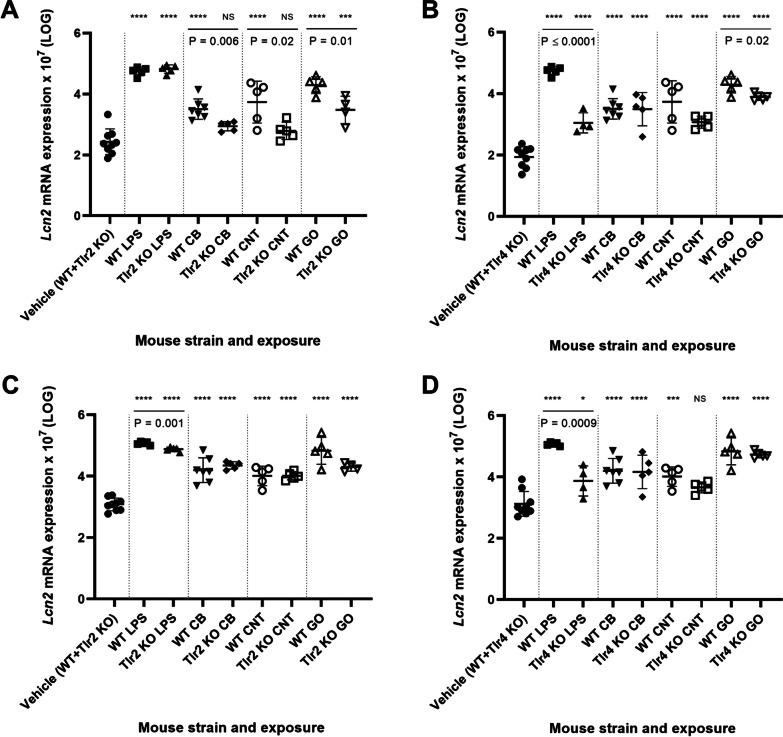
Hepatic mRNA expression levels of *Lcn2* in *Tlr2* KO mice versus WT mice (**A**) and *Tlr4* KO mice versus WT mice (**B**). Pulmonary mRNA expression levels of *Lcn2* in *Tlr2* KO mice versus WT mice (**C**) and *Tlr4* KO mice versus WT mice (**D**). All values are log transformed and presented as mean ± SD. A marker (*) denotes P ≤ 0.05, (**) P ≤ 0.01, (***) P ≤ 0.001, (****) P ≤ 0.0001 compared to vehicle control (Dunn’s multiple comparison method). The vertical lines mark statistically significant differences between KO mice and WT mice (unpaired t-test)

The decreased hepatic *Saa1* mRNA levels were similar to the pattern seen for hepatic NM-induced *Lcn2* mRNA expression (Fig. 8A, B), which also seemed to be TLR2-dependent. The expression pattern of hepatic *Lcn2* and *Saa1* correlated closely across exposures and *Tlr* status (r = 0.9407, p < 0.0001) (Additional file 1: Fig. S9). Thus, hepatic *Saa1* and *Lcn2* expression was dependent on TLR2 for CB, CNT and GO, whereas LPS-induced hepatic *Saa1* and *Lcn2* expression was TLR4-dependent.

The pulmonary levels of *Saa1* and *Lcn2* mRNA (Figs. 6C, D, 8C, D) were related to the hepatic expression of the same genes to assess the possible role of these in the signaling from lung to liver. However, whereas NM-induced hepatic *Saa1* and *Lcn2* expression was TLR2-dependent, lung *Saa1* and *Lcn2* expression were unaffected by TLR2 status, suggesting that the here investigated pulmonary acute phase response genes do not have a strong role in mediating the hepatic acute phase response.

All results are compiled in overview Table 4.

**Table 4:**
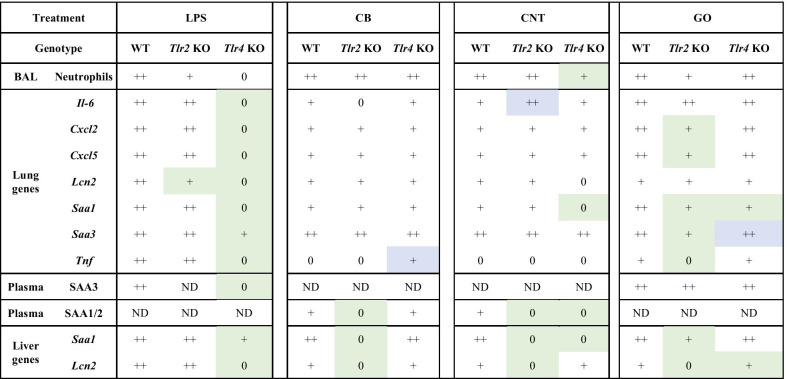
Overview of all results

The markings (0, +, ++) relates to the responses comparing the NM-exposed mice to the corresponding vehicle-exposed, where 0 designates no effect, + indicates statistically significant changes and ++ indicates strong statistically significant changes compared to the corresponding vehicle-exposed group

The green marking indicates a statistically significantly lower response in KO mice compared to WT mice

The blue marking indicates a statistically significantly higher response in KO mice compared to WT mice

*ND* not determined

## Discussion

The aim of the present study was to investigate the interaction between carbon-based NMs and the lung in relation to inflammatory signaling and acute phase response. In addition, we wanted to assess whether the Toll-like receptors TLR2 and TLR4, two crucial cell surface receptors of the innate immune system, are required to initiate the pulmonary and systemic inflammatory response to pulmonary deposited, carbon-based NMs. We used three different NMs, CB, CNT and GO to elucidate the impact of shape and compared the inflammatory responses with that of the well-characterized LPS, which is known to be mediated by TLR4. To this end, we assessed pulmonary inflammation and acute phase response in the lung as well in the liver of *Tlr2* and *Tlr4* KO mice exposed to NMs and LPS as a positive control. The effects were compared to observations in WT mice with the same genetic background.

Optimally, we would have included more doses and time points, but due to costs and limited availability of the *Tlr4* KO-model from the supplier, we had to limit the study design to a single dose and time point in order to be able to include three different NMs. However, we have previously shown that the acute phase response in the liver is present at 24 h post-exposure, but not at later time points even for MWCNT [33]. As for the pulmonary response, we believe that the 24 h time point is the most well characterized acute time point in terms of neutrophil influx and gene expression [29, 33–36, 55, 56].

Due to improvement of statistical power and due to the differences in group-sizes, we chose to combine the vehicle-exposed controls for WT mice and *Tlr2* KO mice in the analysis of TLR2-related effects, and WT mice and *Tlr4* KO mice in the analysis of TLR4-related effects. There were no statistically significant differences between the control groups for any outcome. However, the comparison of the endpoints across mouse strains were independent of the vehicle-exposed control groups.

As expected, *Tlr4* KO mice exposed to LPS generally showed a reduced inflammatory response indicated by a 70% reduction of neutrophil influx. Compared to WT mice, LPS-exposed *Tlr2* KO mice showed reduced levels only for lung *Lcn2* gene expression. Moreover, LPS-exposed *Tlr4* KO mice did not show any pulmonary cytokine/chemokine responses (*Il-6*, *Tnf, Cxcl2, Cxcl*5 mRNA) and no increase of systemic SAA3 protein levels in plasma. However, for other genes, such as *Saa3* in the lungs and *Saa1* mRNA in the liver, the LPS-response in *Tlr4* KO mice remained significantly increased over the vehicle control, yet still lower than the corresponding WT mice, suggesting that TLR4-independent mechanisms, such as via TLR2, may partly regulate their LPS-sensitive gene expression.

The neutrophil influx has been shown to peak for LPS- and CB-induced inflammation around 12–24 h after exposure and declines rapidly over time [45, 57]. For practical experimental reasons, 24 h was preferred as the single time point for the investigation of the acute inflammatory response, even though we have previously shown that CNT- and GO-induced pulmonary inflammation rises over time and is higher 3 days post-exposure [34, 35]. The selected dose levels aimed to reach similar levels of roughly 100.000 neutrophils (total recovered cells per lung) in WT mice when exposed to 162 µg CB, 54 µg CNT, and 18 µg GO. The factual levels of neutrophils differed slightly with neutrophil mean levels about 132.000 for CB, 71.000 for CNT and 218.000 for GO, but this had no practical impact as the differences between type of exposure are not directly compared in the study. The dose levels are representative for the potential human exposure in occupational settings. Based on the current Danish occupational exposure limit of 3.5 mg/m^3^ and the lung deposition reported as lung burden in mice by Heinrich et al. [58], a dose level of 162 µg/mouse CB would correspond to the retained lung burden after 3 months of occupational exposure to CB. Noteworthy, much higher occupational exposure levels (14.9 mg/m^3^) have been reported [59]. There are currently no occupational exposure limit for CNTs and GO.

Although, the measurement of mRNA expression does not necessary parallel the production of bioactive proteins, we consider qRT-PCR a reliably method and thus interpret increased mRNA expression to represent increased protein expression. Previous publications demonstrated that plasma SAA3 [25, 32, 33] and SAA1 [33] protein levels correlated with mRNA expression in lung tissue of CNT-exposed mice. Following pulmonary CB exposure, we have previously reported dose-dependent increased protein levels of the following cytokines: IL1β, IL2, IL3, IL5, IL6, IL9, IL10, IL12B, IL13, IL17, CCL2, CCl4, CXCL1, CXCL2, INFG and TNF on lung tissue [60]. Occupationally CB-exposed workers had elevated blood levels of IL1β, IL6, IL8, MIP1β and TNF [59]. Thus, the CB-induced pulmonary gene expression pattern fits well with previously reported blood levels of cytokines in mice and humans.

We have consistently found that pulmonary exposure to NMs by inhalation or by instillation induces a pulmonary acute phase response characterized by induced levels of SAA3 [20]. SAA are pleotropic proteins that also function as monocyte- and granulocyte chemoattractants [61] and the *Saa3* mRNA levels in lung tissue correlates with the number of neutrophils in BAL fluid in several in vivo studies [25, 33, 47, 48, 50, 51]. In the present study, the pulmonary gene expression of *Saa1*, *Saa3* and *Lcn2*, which are all among the top 10 ranked acute phase response genes in lung tissue of mice after exposure to various NMs by i.t. instillation [20], correlate with neutrophil levels.

Here, we provide evidence that the NM-induced inflammatory and acute phase response differ from the LPS-mediated response. LPS exposure at moderate levels, not provoking significant tissue injury, caused a strong induction of pro-inflammatory cytokines *Il-6* and *Tnf* mRNA levels, which were almost reduced to control levels in *Tlr4* KO mice. This pattern were paralleled by lowered expression levels of *Saa3*, *Saa1*, *Lcn2*, *Cxcl2*, *Cxcl5* and a numerically lowered neutrophil influx in LPS-exposed *Tlr4* KO mice. In addition, systemic, serum SAA3 and hepatic acute phase response in terms of *Saa1* and *Lcn2* expression levels was also absent in *Tlr4* KO mice after LPS exposure.

CB-mediated pulmonary inflammation was virtually unaffected by TLR2 and TLR4 status, although a tendency of decreased *Il-6* and *Tnf* expression was observed in *Tlr2* KO mice. CB caused comparable neutrophil levels in all mouse strains, indicating no essential contribution of TLR2 or TLR4 signaling to the acute inflammatory response. Overall, the results suggest that CB-mediated pulmonary inflammation is independent of TLR2 and TLR4.

CNT-mediated pulmonary inflammation was most affected in *Tlr4* KO mice, in which the BAL neutrophil influx and macrophage cell numbers were diminished, compared to WT mice. *Saa1* mRNA expression was reduced in *Tlr4* KO mice exposed to CNT, but levels failed to show significant increases over vehicle controls. The latter may be seen in agreement with previous findings that *Saa1* expression correlates closely with neutrophil influx following NM-exposure [20].

The neutrophil influx was 35% decreased in GO-exposed *Tlr2* KO mice, although this was a non-significant observation, most of the analyzed lung markers *Tnf*, *Saa3*, *Saa1*, *Lcn2*, *Cxcl2* and *Cxcl5* were significantly decreased as compared to WT mice. Only the levels of *Il-6* and *Lcn2* mRNA in the lung and plasma SAA3 remained unaffected by the *Tlr2* genotype in GO-exposed mice. Overall, the results suggest an essential involvement of TLR2 in the response to GO. This may suggest a direct interaction between GO and TLR2, since the other NMs induce similar expression patterns for *Saa1* and *Saa3*, which are known to interact directly with TLR2 [18, 62]. The studied GO has a very low C/O ratio, suggesting a heavily hydroxylated surface [63]. This may have a structural resemblance to the polysaccharide part of LPS. In agreement with this, GO induces a very strong acute phase response following pulmonary exposure in mice as compared to reduced GO, which contains much less oxygen [35].

In summary, our data clearly demonstrate, that TLR2 or TLR4 signaling is not fully required for the initiation of the acute pulmonary inflammatory response. TLR2 contributed essentially to the response to GO exposure and TLR4 to that of CNTs. However, if these carbon structures may specifically bind to TLR2 and TLR4 complexes expressed on various lung cells, this interaction is not required to drive the NM-triggered pulmonary inflammation. However, the fiber-like structural resemblance between LPS [64] and the thick and straight CNT used in the current study could suggest that the CNT interacts directly with TLR4, as suggested by computational studies [26, 27]. The three carbon-based NMs differ by shape, but also by surface chemistry. The CB Printex 90 from Evonik/Degussa consists of more than 99 wt% elemental carbon with traces of organic compounds (less than 1%). We have previously reported that TEM analysis showed that Printex 90 in instillation suspension consisted of both free and open to partially open chain-agglomerates and that high-resolution analysis showed that the spheres consisted of concentric layers with graphitic spacing, resembling nano-onions structures [29]. The Mitsui-7 CNT has a low oxygen content [65] and therefore presumably a hexagonal lattice structure on the surface, whereas the GO used in this study has a very low C/O ratio suggesting a highly hydroxylated surface as compared to the unmodified CNT [63]. It is possible that both shape and surface chemistry are important determinants for the molecular interaction with PRRs.

The observed differences in the contribution of TLR2 or TLR4 signaling to the inflammatory response could also reflect that the different NMs interact with different cell types in the lungs, or TLR-ligands, such as DAMPs released from the CNT- or GO-exposed cells enhance the elicited inflammation. We have recently shown that LPS and CB exposure induces *Saa3* expression in different cell types in the lungs of mice using single cell sequencing [22]. Thus, LPS-exposure induced *Saa3* expression predominantly in non-classical monocytes, whereas CB induced *Saa3* expression in mesenchymal cells [22]. We previously also provided evidence, that in contrast to LPS, CB exposure rather stimulates lung epithelial cells, whereas resident lung macrophages remain quite quiescent [45]. Our current data again support the notion that pulmonary inflammation signaling differs substantially between LPS and the here tested NMs, and between the different NMs.

Most interestingly, we observed the strongest TLR involvement in the induction of hepatic acute phase response characterized by hepatic *Saa1* and *Lcn2* expression after pulmonary NM-exposure. The observed NM-dependent hepatic acute phase response is in concordance with previous findings for the same or analogue materials [29, 32, 35, 36], as well as for other CNTs. NM translocation from lung to liver has been demonstrated for CB [66], MWCNT [67, 68] and few layer graphene [69]. NMs accumulate primarily in Kupffer cells in the liver [70]. Histopathological examination of the liver has been included in several studies. The MWCNT NM-401, with similar physicochemical properties as Mitsui-7, was shown to induce histopathological changes in the liver 1 day post-exposure, with an increase in lesion frequency 3 and 28 days post-exposure [32]. These changes included vacuolar degeneration, granulomas, necrosis of hepatocytes, and increased number and/or hypertrophy of Kupffer cells, all indicative of inflammation and general hepatic pathology. Similar hepatic histological changes were observed after pulmonary exposure to nano-sized CB and nanosized TiO_2_ [56]. The observed LPS-mediated hepatic response was dependent on TLR4 and independent of TLR2, as was the level of pulmonary inflammation. LPS-induced liver inflammation has been shown to depend on TLR4 when comparing WT and TLR4−/− mice exposed to LPS and similar responses were provoked in primary hepatic Kupffer cells isolated from the mice following LPS exposure [71]. This suggests that the TLR4-dependent liver inflammation was initiated by a direct interaction between LPS and Kupffer cells. It is possible, that translocated MWCNT also interacts directly with TLR4 on Kupffer cells. Similarly, it is possible that the three different NMs interact directly with TLR2 on Kupffer cells following translocation, and that this forms the basis of the observed differences in TLR-dependency of the hepatic acute phase response.

## Conclusions

In conclusion, the involvement and requirement of TLR2 or TLR4 signaling for lung inflammation and lung and liver acute phase response differed between the three studied NMs, indicating that shape and surface chemistry may be determinants of the pathways triggered even at comparable levels of pulmonary inflammation. Particularly, these differences led to significant differences in the downstream systemic signaling as indicated by the observed differences in TLR2- and TLR4-dependency of the expression of hepatic acute phase response genes. In the lung, the GO-induced inflammation was to a considerable degree dependent on TLR2, and MWCNT-induced inflammation was partly TLR4-dependent. From this data, it could be suggested, that TLR2 is either involved in the pulmonary sensing of the dosed GO, and TLR4 in the sensing of the specific MWCNTs (Mitsui-7), or that NM specific injury pattern cause a specific release of DAMPs, functioning as TLR2 or TLR4 ligands, respectively. However, even though a direct NM-TLR interaction could not be studied here, our data clearly show that the innate immune response receptors TLR2 nor TLR4 are not requisite for the acute lung toxicity of CB, but contribute to that of the specific GO and CNT investigated here. TLR2 was required for the induction of acute phase response in the liver for all three NMs, whereas LPS-dependent signaling to the liver was entirely TLR4-dependent. The TLR4-dependent response in liver has been shown to depend on direct interaction between Kupffer cells and LPS. TLR4 was also required for induction of MWCNT-induced *Saa1* gene expression in the liver, but not for liver *Saa1* expression triggered by CB or GO exposure. The difference in TLR-dependency may suggest that the liver response were induced by direct interaction with translocated NMs, but this warrants further investigation.

## Methods

### Nanomaterials and positive control

The NMs included CB, multi-walled CNT and GO. The CB (Printex 90) was a kind gift from Degussa-Hüls, Frankfurt, Germany. CB has been extensively studied, and used as a benchmark particle in previous in vivo studies [29, 30, 33, 47, 51, 55, 72]. The characterization of CB has been described in detail elsewhere [29, 73]. GO was delivered in a water suspension manufactured and supplied by Graphena (San Sebastian, Spain). A detailed description and characterization of GO has been published previously [63]. The CNT was the multi-walled Mitsui 7 was a kind gift from Hadoga Chemical industry, Japan (former Mitsui Company) and the characterization has also been published previously [65]. LPS was included as a positive control (Lipopolysaccharides from Escherichia Coli O55:B5, Sigma-Aldrich).

### Preparation of nanomaterials for exposure and characterization

The NMs were suspended in 2% v/v C57BL/6 mouse serum in Nanopure Diamond water at a final concentration of 3.24 mg/ml and probe sonicated on ice, for 16 min with 10% amplitude without pause, using a Branson Sonifier S-450D (Branson Ultrasonics Corp., Danbury, CT, USA) equipped with a disruptor horn (model number 101-147-037) as previously described [74]. For the vehicle control group, 2% v/v C57BL/6 mouse serum in Nanopure Diamond water was similarly sonicated.

The average hydrodynamic particle size of the NMs in instillation suspensions (3.24 mg/ml) were measured by Dynamic Light Scattering ((DLS); Malvern Nano Zetasizer equipment mounted with a 633 nm red laser)). The results were obtained from six repeated analyses of the same sample.

### Animal handling and exposure

Seven-week-old female mice (*Tlr2* KO mice, B6.129-*Tlr2*^*tm1Kir*^/J, stock 004650), (*Tlr4* KO mice, B6(Cg)-*Tlr4*^*tm1.2Karp*^/J, stock 029015), and C57BL/6 J (wildtype (WT) mice, stock 000664) were purchased from The Jackson Laboratory (Bar Harbor, Maine, USA). The mice were randomized to polypropylene cages and had access to food (Altromin 1324, Christian Petersen, Denmark), water ad libitum and to Solid Drink® Standard Bio with hydrocolloids > 98% water during the first days after arrival. The housing conditions have been described in detail elsewhere [47]. At 8 weeks of age, the mice were anaesthetized and exposed to NMs, LPS or vehicle by single i.t. instillation as described [75]. The group sizes varied (N = 4–7 per group) due to the unexpected death of a few mice upon arrival, limited availability of KO mice and reordering of new mice to be included in the latter part of the experiment to counteract these difficulties. Following sonication of the exposure stock as described above, the NM suspensions were diluted to obtain final concentrations of 18 µg GO, 54 µg CNT and 162 µg CB per 50 µl, which was the instillation volume per mouse, and sonicated again for 2 min prior instillation. LPS was diluted in sterile 0.9% sodium chloride to the final concentration of 4 µg per 50 µl. The vehicle control mice received 50 µl of Nanopure Diamond water added 2% v/v C57BL/6 mouse serum. The doses of the NMs were based on previous in vivo studies, where the influx of neutrophils reached levels around 100.000 [35, 37] and therefore the doses differed between the types of NM. The instillation procedure has been described in detail previously [73, 75]. All animal procedures followed the guidelines according to the EC Directive 86/609/EEC and the Danish law. The experiments were approved by the Danish “Animal Experiment Inspectorate” under the Danish Ministry of Justice (2015-15-0201-00465).

### Collection of BAL cells, plasma and tissue

Mice were anesthetized and necropsied 1 day post-exposure. To obtain the bronchoalveolar (BAL) fluid the lungs were flushed twice with sterile 0.9% sodium chloride through the trachea. The BAL fluid was kept on ice until separation of fluid and cells by centrifugation at 400×*g* for 10 min at 4 °C. Heart blood was withdrawn via intracardiac puncture and stabilized with K_2_EDTA. It was then fractionated by centrifugation and plasma was collected and stored at − 80 °C. Small pieces of lung and liver tissues were snap frozen in cryotubes in liquid nitrogen and stored at − 80 °C until isolation of RNA for mRNA expression analysis.

### Differential counting of BAL cells

BAL cell composition was determined as previously described [74]. In short, the separated BAL cells were resuspended in 100 µl HAMF12 medium containing 10% fetal bovine serum. 40 µl of the cell suspension was mixed with 160 µl medium containing 10% dimethyl sulfoxide (DMSO). The total number of cells and of dead cells was determined from 20 µl diluted cell suspension by NucleoCounter NC-200TM (Chemometec, Allerød, Denmark). 40 µl of the cell suspension was collected on microscope slides by centrifugation at 60×*g* for 4 min and where after the cells were fixed with 96% ethanol and stained with May–Grünwald–Giemsa. The samples were randomized and blinded before scoring of macrophages, neutrophils, lymphocytes, eosinophils, and epithelial cells, which were assessed by counting 200 cells/sample under light microscope (100× magnification).

### Measurement of mRNA expression levels

Gene expression levels was measured by quantitative real-time reverse transcriptase polymerase chain reaction (RT-PCR) as previously described [76]. In short, the acute phase response was assessed by the measurement of *Saa3*, *Saa1* and *lipocalin 2 (Lcn2)* mRNA expression levels in lung and/or liver tissue. Inflammation was assessed by the measurement of *interleukin-6 (Il-6)*, *tumor necrosis factor (Tnf)*, *chemokine ligand 2 (Cxcl2)* and *chemokine ligand 5 (Cxcl5)* mRNA expression levels in lung tissue. Total RNA was isolated using Maxwell® 16 LEV simply RNA Tissue Kit (AS1280, Promega, USA) according to the manufacturer protocol. Complementary DNA (cDNA) was prepared using TaqMan® reverse transcription reagents (Applied Biosystems, USA) according to the manufacturer protocol. Total RNA and cDNA concentrations were measured on NanoDrop 2000c (ThermoFisher, USA). The gene expression levels was determined using predesigned TaqMan expression assays and 18S rRNA as endogenous control (Applied Biosystems, USA). The samples were run in triplicates using ViiA7 Real-Time PCR detector (Applied Biosystems, USA). Negative controls were included in each run of the analysis. The relative expression of the target gene was measured by the comparative C_T_ method after thoroughly assay optimization and validation.

### Measurement of total protein in BAL fluid

Total protein content in BAL fluid was measured by Pierce™ BCA Protein Assay Kit (Thermo Scientific, USA) according to the manufactures protocol and has been described in detail by [55].

### Measurement of SAA3 protein in plasma

The SAA3 protein levels were determined in plasma and carried out by sandwich ELISA in accordance with the manufacturer’s instructions (Mouse Serum Amyloid A-3, Cat.#EZMSAA3-12K, Millipore) and has been described in detail by [33]. Only *Tlr2* KO mice exposed to vehicle and GO and *Tlr4* KO mice exposed to vehicle, LPS and GO were analyzed due to the cost of the kits.

### Measurement of SAA1/2 protein in plasma

Plasma levels of serum amyloid A1 and A2 (SAA1/2) protein were measured using the Tridelta PHASE™ Murine Serum Amyloid A ELISA Assay (BioRépair, Sinsheim, Germany) according to the manufacturer’s instructions. Due to experimental failures, one ELISA plate was discarded. The remaining measurements included all samples from CB and CNT exposed mice as well as vehicle samples from three WT mice and two *Tlr4* KO mice, which were consequently pooled in the statistical analysis.

### Statistical analysis

Statistical analyses were performed using the software package Graph Pad Prism 7.02 (Graph Pad Software Inc., La Jolla, CA, USA). In the statistics, we combine the vehicle-exposed controls for WT mice (n = 4) and *Tlr2* KO mice (n = 5) in the analysis of TLR2-effects, and vehicle-exposed WT mice (n = 4) and *Tlr4* KO mice in the analysis of TLR4-effects (n = 5). There were no statistically significant differences between the control groups for any outcome. All data are expressed as mean ± standard deviation and were tested for normality using the Shapiro-Wilks test and for variance homogeneity using the Brown-Forsythe test. The BAL cell count data did not fulfill normality and variance homogeneity criteria and the data were analyzed by the nonparametric Kruskal–Wallis test to test the differences between the exposure groups and the vehicle, and the Mann–Whitney test to test the differences between WT and KO mice. Gene expression data were logarithmically transformed and analyzed by Dunnetts’s multiple comparison method to test the differences between the exposure groups and the vehicle control group, and the unpaired t-test to test the differences between WT and KO mice. Total protein and the levels of SAA3 were analyzed by Dunnetts’s multiple comparison method to test the differences between the exposure groups and the vehicle, and the unpaired t-test to test the differences between WT and KO mice. The SAA1/2 protein levels were analyzed by the nonparametric Kruskal–Wallis test to test the differences between the exposure groups and the vehicle, and the Mann–Whitney test to test the differences between WT and KO mice. P-values ≤ 0.05 was considered significant. The Spearman rank correlation test was used to analyze the association between influx of neutrophils and acute phase response in lung tissue.

## Supplementary Information

**Additional file 1.** Description of the pilot studies using Sparstolonin B.

## Data Availability

The datasets analyzed during the present study are available from the corresponding author on reasonable request.
