# Supplementary material for: Nanomaterial- and shape-dependency of TLR2 and TLR4 mediated signaling following pulmonary exposure to carbonaceous nanomaterials in mice
Source: Part Fibre Toxicol. 2021 Oct 30;18:40. doi: 10.1186/s12989-021-00432-z (PMC8557558; doi:10.1186/s12989-021-00432-z)
Supplement: Supplementary file 1 — Additional file 1. Description of the pilot studies using Sparstolonin B. [file 12989_2021_432_MOESM1_ESM.docx]

**Description of the pilot studies using Sparstolonin B**.

**PILOT I:** In the first pilot study, we wanted to verify that one type of commercially available TLR-antagonist, Sparstolonin B (Sigma-Aldrich, Merck) was able to inhibit acute LPS-mediated lung inflammation in mice. We were aiming to optimize the experimental conditions by 1) using different concentrations of Sparstolonin B and 2) give a repeated dose after the initial exposure.

Sparstolonin B attenuates the interaction of MyD88 and TLR2 and TLR4/IL-1 receptor domain and inhibits both TLR2 and TLR4 Myd88 dependent function, where TRIF, TRAM dependent IRF3-activation is not affected.

The TLR-antagonist was administered to female C57BL/6 mice (Taconic, Ejby, Denmark) (n=7) by intraperitoneal (i.p.) injection 15 minutes before exposure to LPS by i.t. instillation. The doses were either 6 or 9 mg/kg Sparstolonin B and 4 µg LPS. Two animal groups received a repeated dose of Sparstolonin B (6 or 9 mg/kg) five hours after the initial exposure. The LPS dose was chosen as a dose where the neutrophil influx would be significantly increased. The antagonists were diluted in 10% DMSO/saline and LPS were dissolved in saline. We included the controls: LPS alone, vehicle, and blank.

The overall result (see figure below) showed that a significantly declined LPS-response was obtained at 9 mg/kg Sparstolonin B and by repeated i.p. injection (9 mg/kg) five hours after initial LPS/Sparstolonin B exposure.


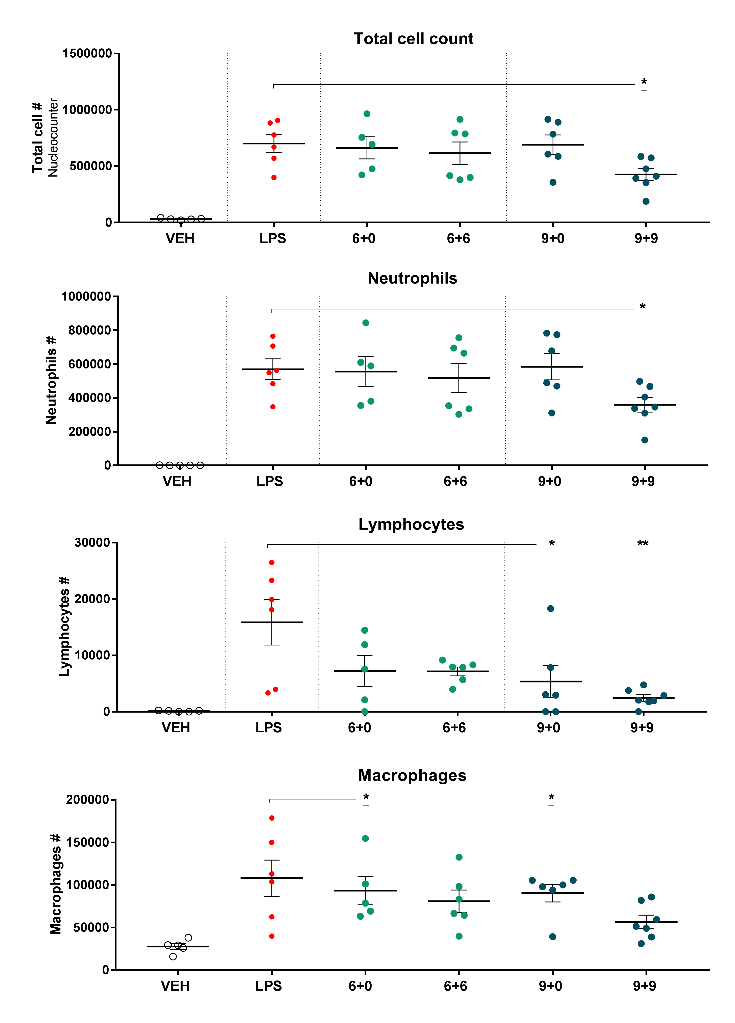


**PILOT II:** In pilot II, we investigated whether the inflammatory response to carbon black (CB, Printex90) and graphene oxide (GO) is affected by TLR2/4 inhibition in a similar manner as observed for LPS. Therefore, we exposed mice with and without administration of 9 mg/ml Sparstolonin B given i.p. 15 minutes before i.t. instillation with 5 µg LPS, 18 µg GO, 162 µg CB and 5 hours after i.t. instillation.

The overall results, as shown on the figures below, show a decrease in the LPS-response (for total cells, neutrophils and lymphocytes). However, there were no difference in the NM-responses with and without administration of Sparstolonin B. In addition, there were no difference in *Saa3* mRNA expression levels in the lung.

We conclude that after optimization of doses and administration time, we show that the LPS-response (influx of immune cells) is significantly decreased after administration of the TLR-antagonist Sparstolonin B. However, we did not see any effect on the NM-response. Thus, this was a first indication that in contrast to LPS, MyD88-IRAK activation, downstream of TLR2 or TLR4 is not required for NM-triggered inflammation.

To further investigate the inflammatory effects and acute phase response associated to TLR2 and/or TLR4 signaling, we conducted the main animal study using *Tlr4* and *Tlr2* knockout mice.


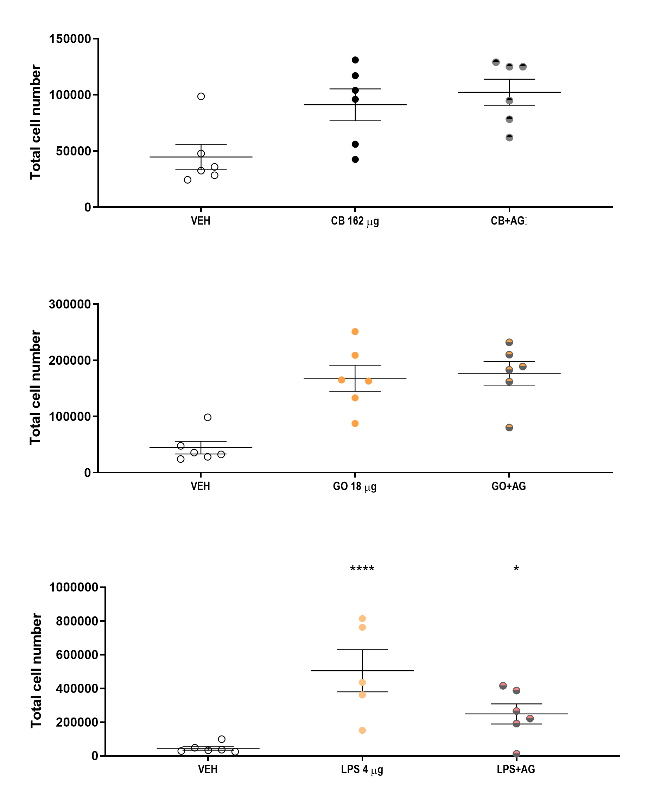

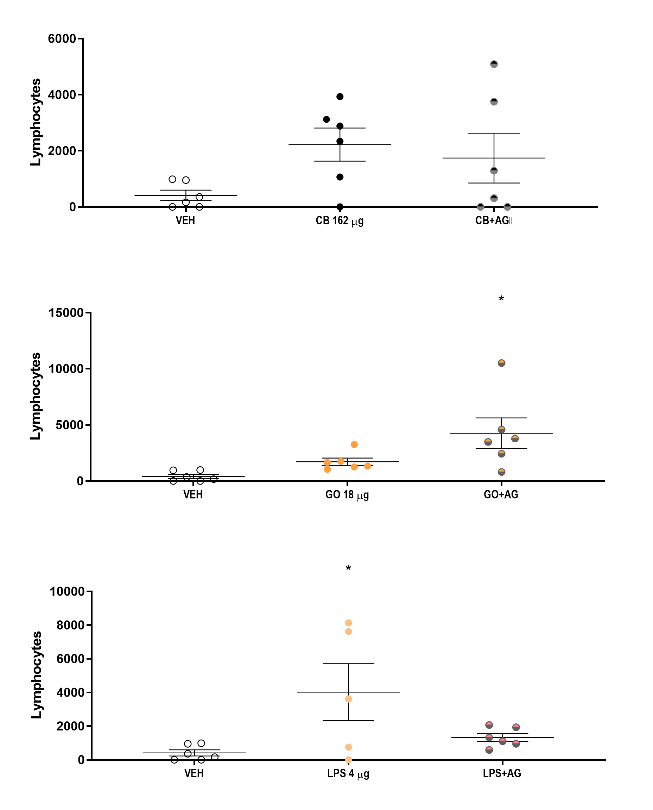


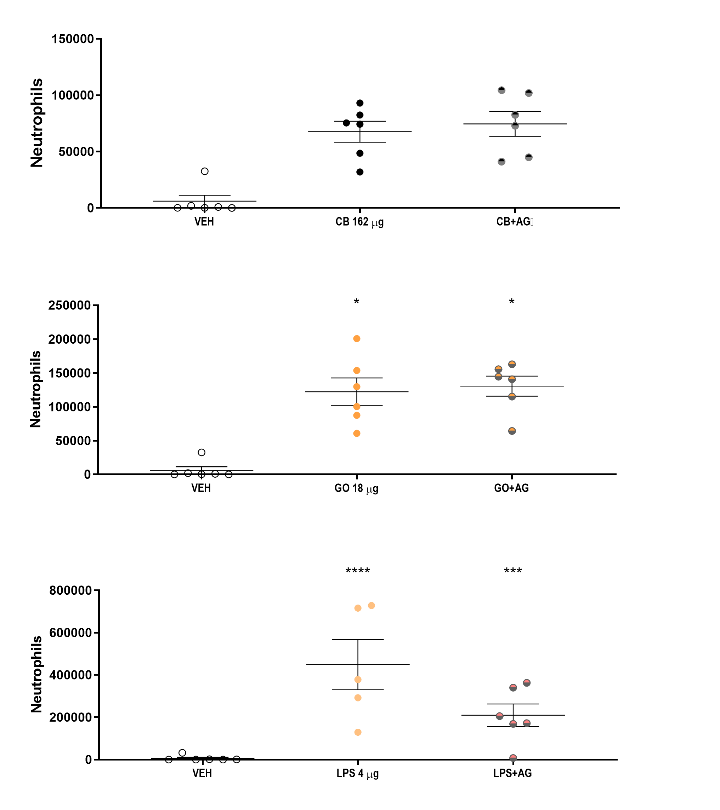

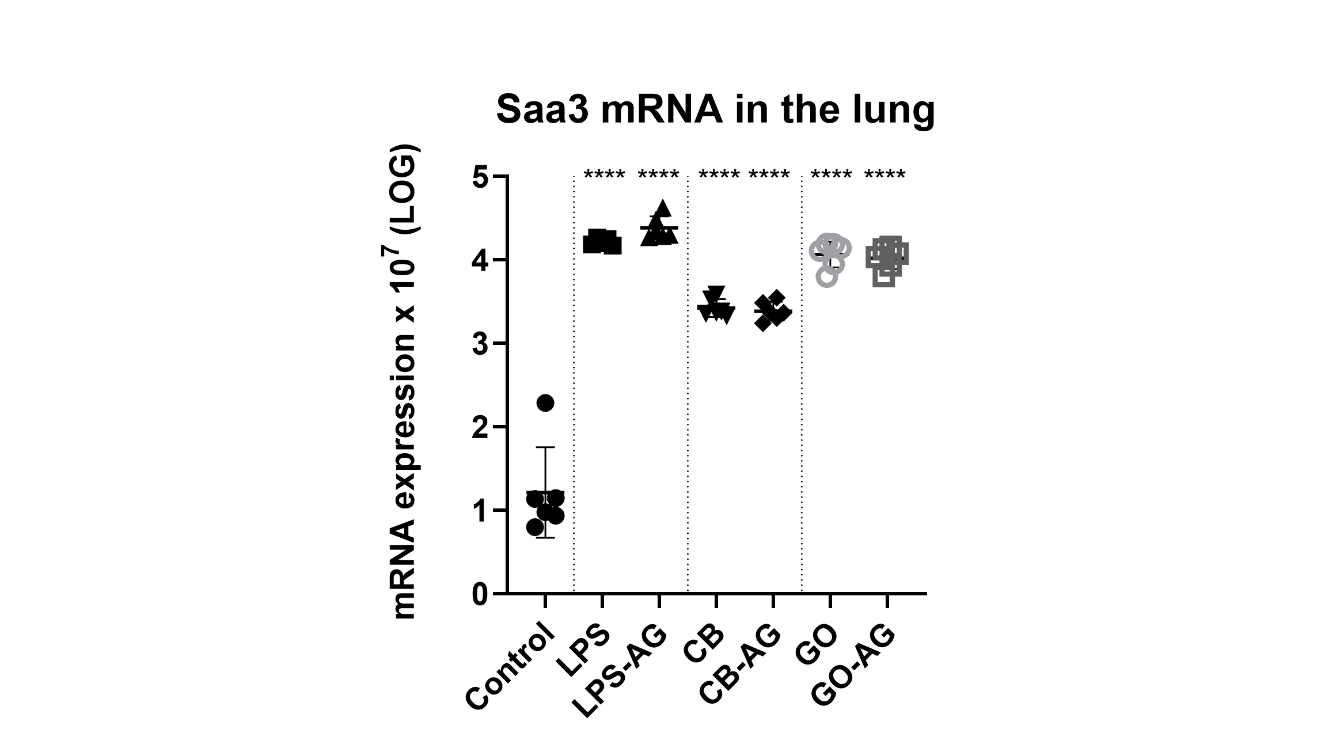


**Table S1**

|  | z-average size | PI |  |
| --- | --- | --- | --- |
| CB | 91.8 nm | 0.177 |  |
| CNT | 601.2 nm | 0.296 |  |
| GO | 166.0 nm | 0.223 |  |

The intensity-based z-average size and polydispersity index (PI) of NMs in instillation suspensions (3.24 mg/ml in Nanopure water with 2 % mouse serum).


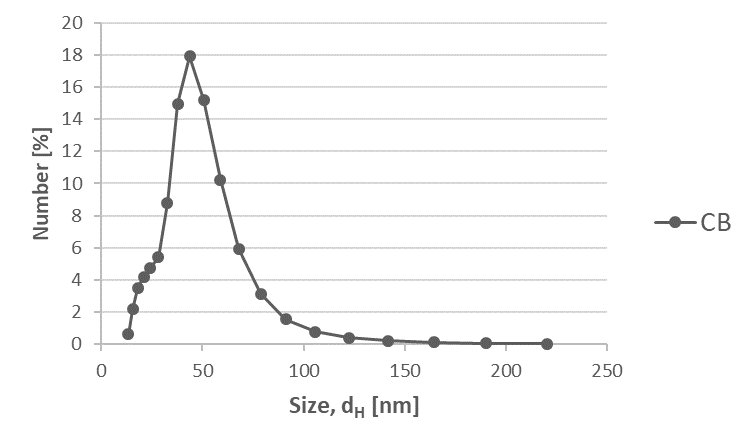

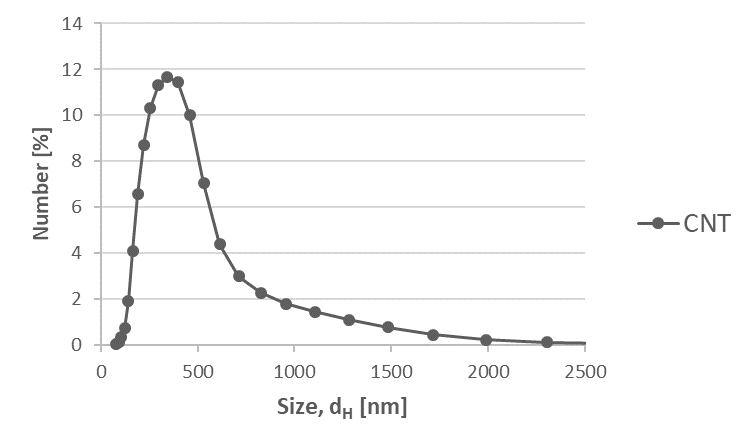

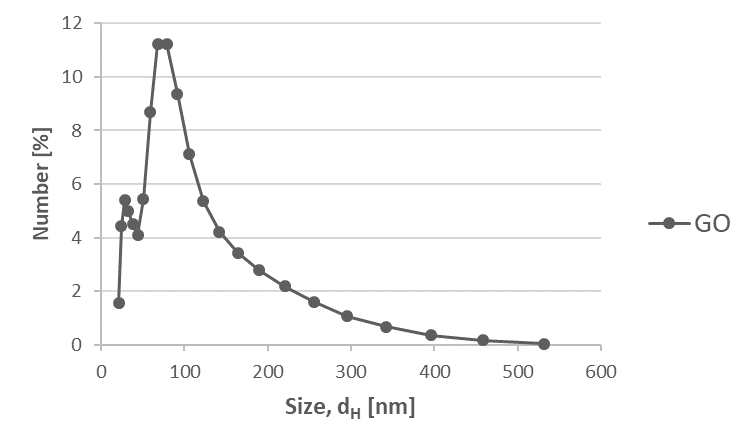


**Fig. S1** Dynamic light scattering number-based size distributions of NMs in instillation suspensions (3.24 mg/ml in Nanopure water with 2 % mouse serum).


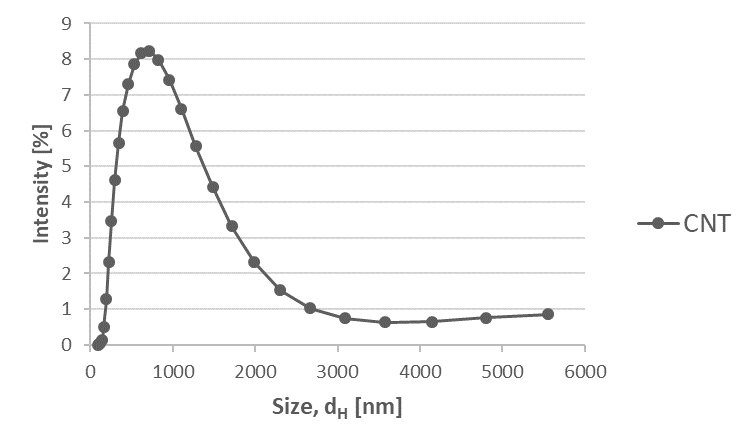

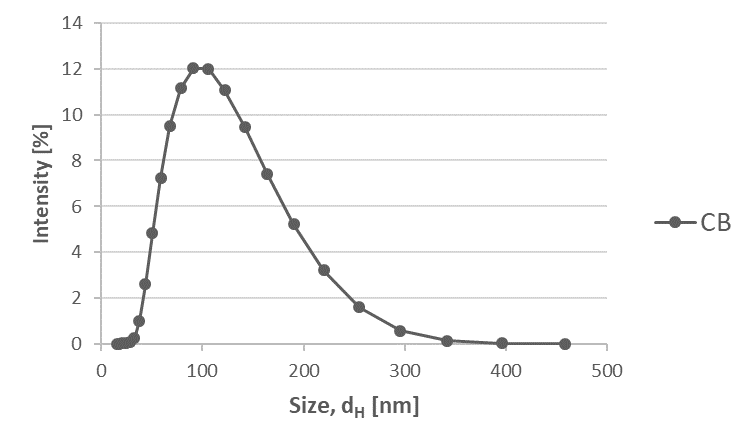

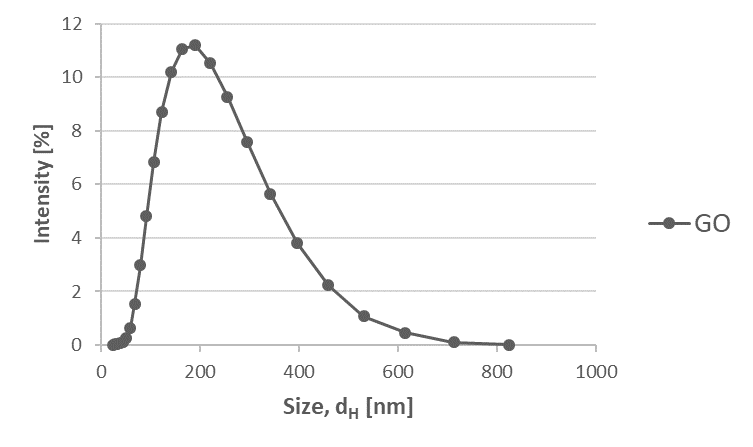


**Fig. S2** Dynamic light scattering intensity-based size distributions of NMs in instillation suspensions (3.24 mg/ml in Nanopure water with 2 % mouse serum).


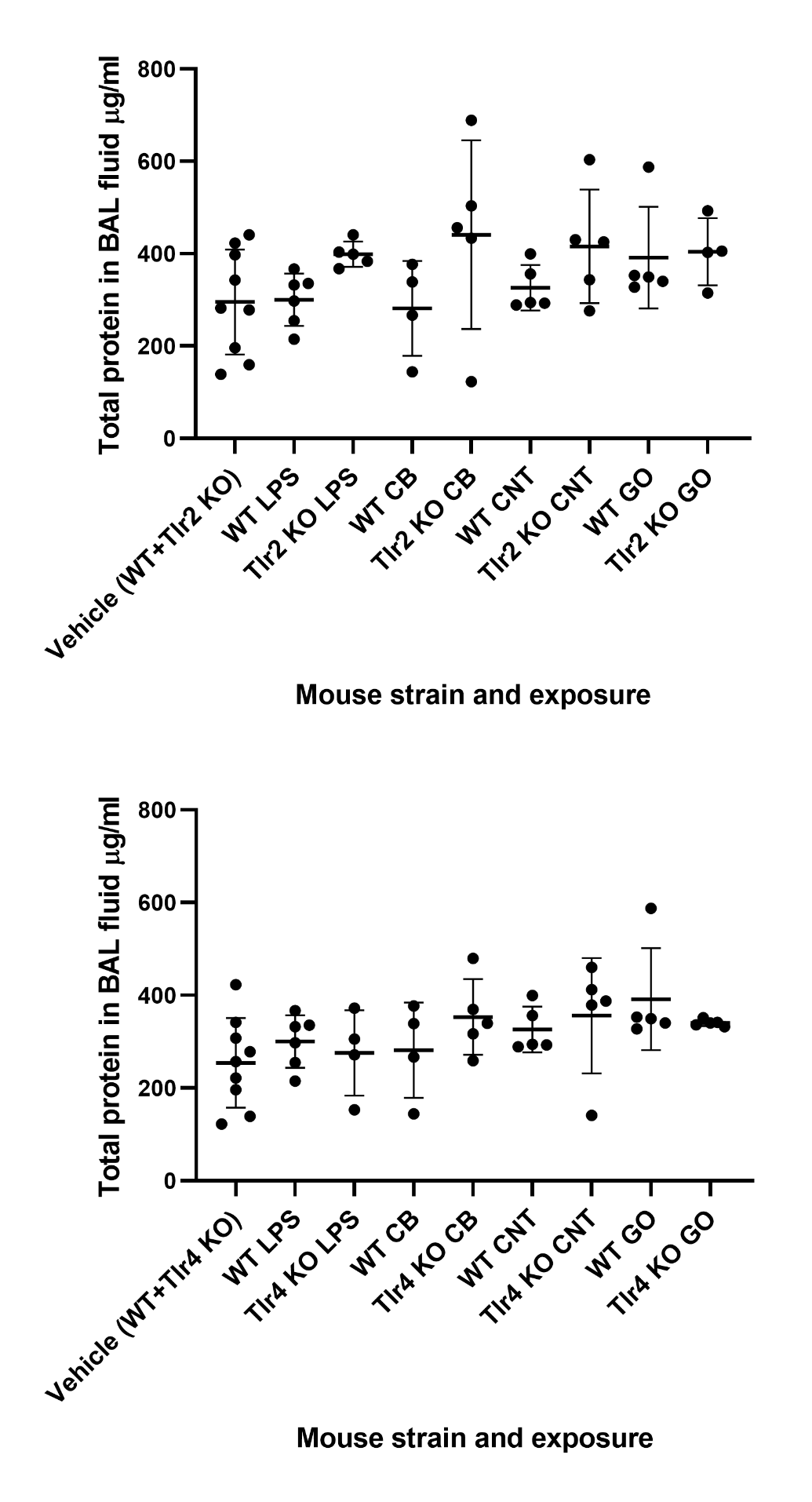


**Fig. S3** Total protein content in BAL fluid.

**
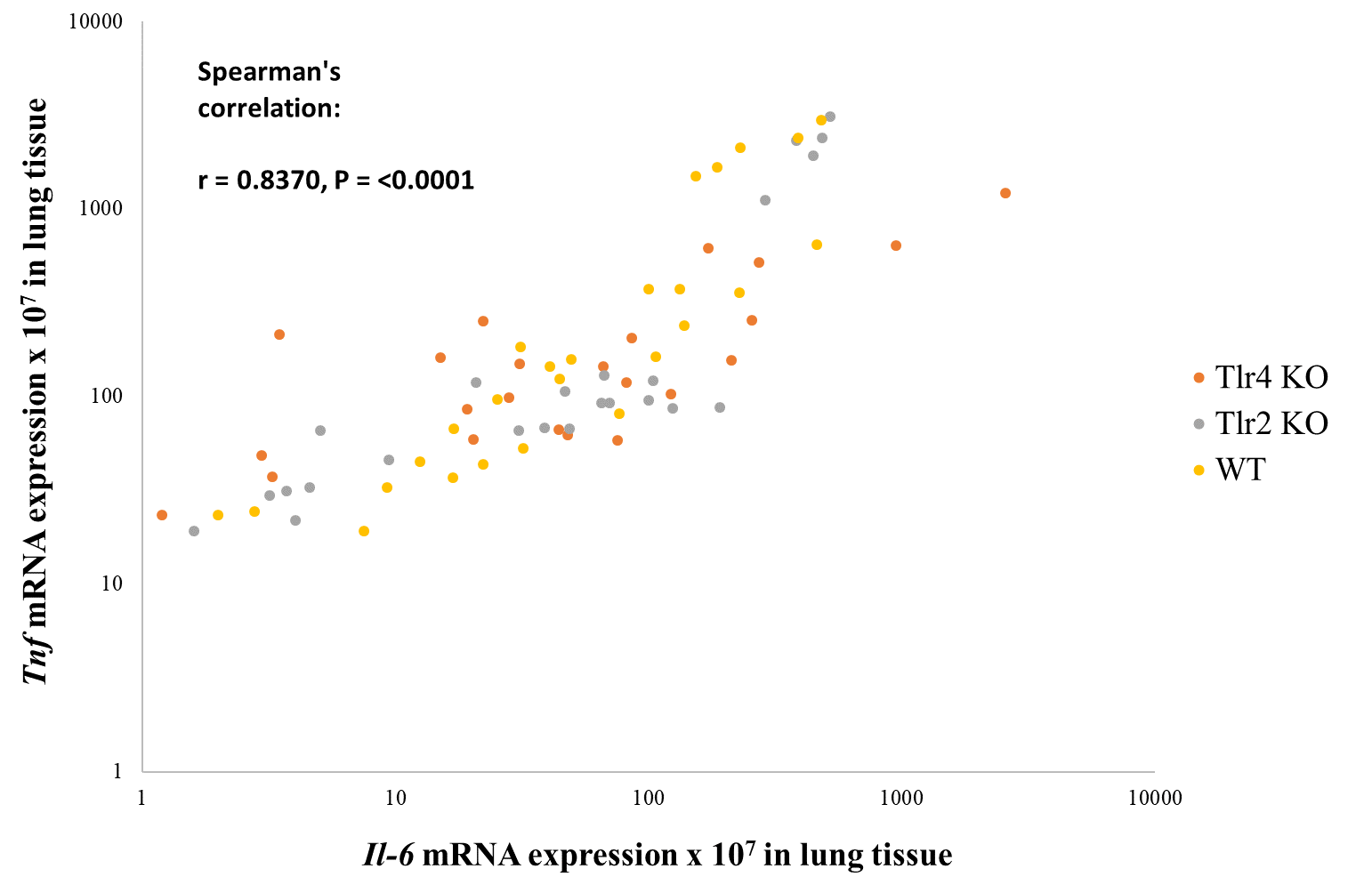
**

**Fig. S4** The correlation between *Il-6* and *Tnf* mRNA levels in lung tissue (double logarithmic scale).


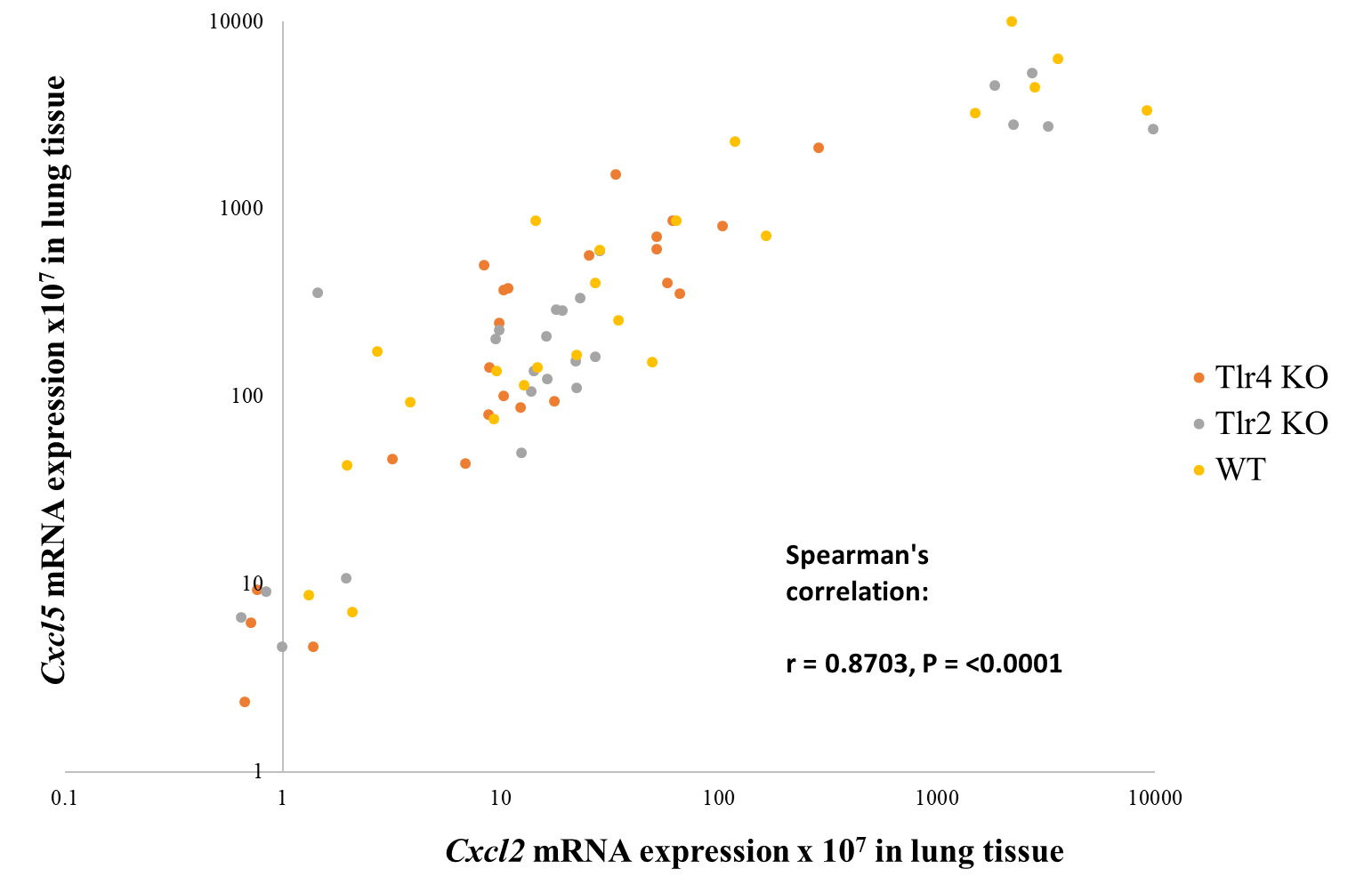


**Fig. S5** The correlation between *Cxcl2* and *Cxcl5* mRNA levels in lung tissue (double logarithmic scale).


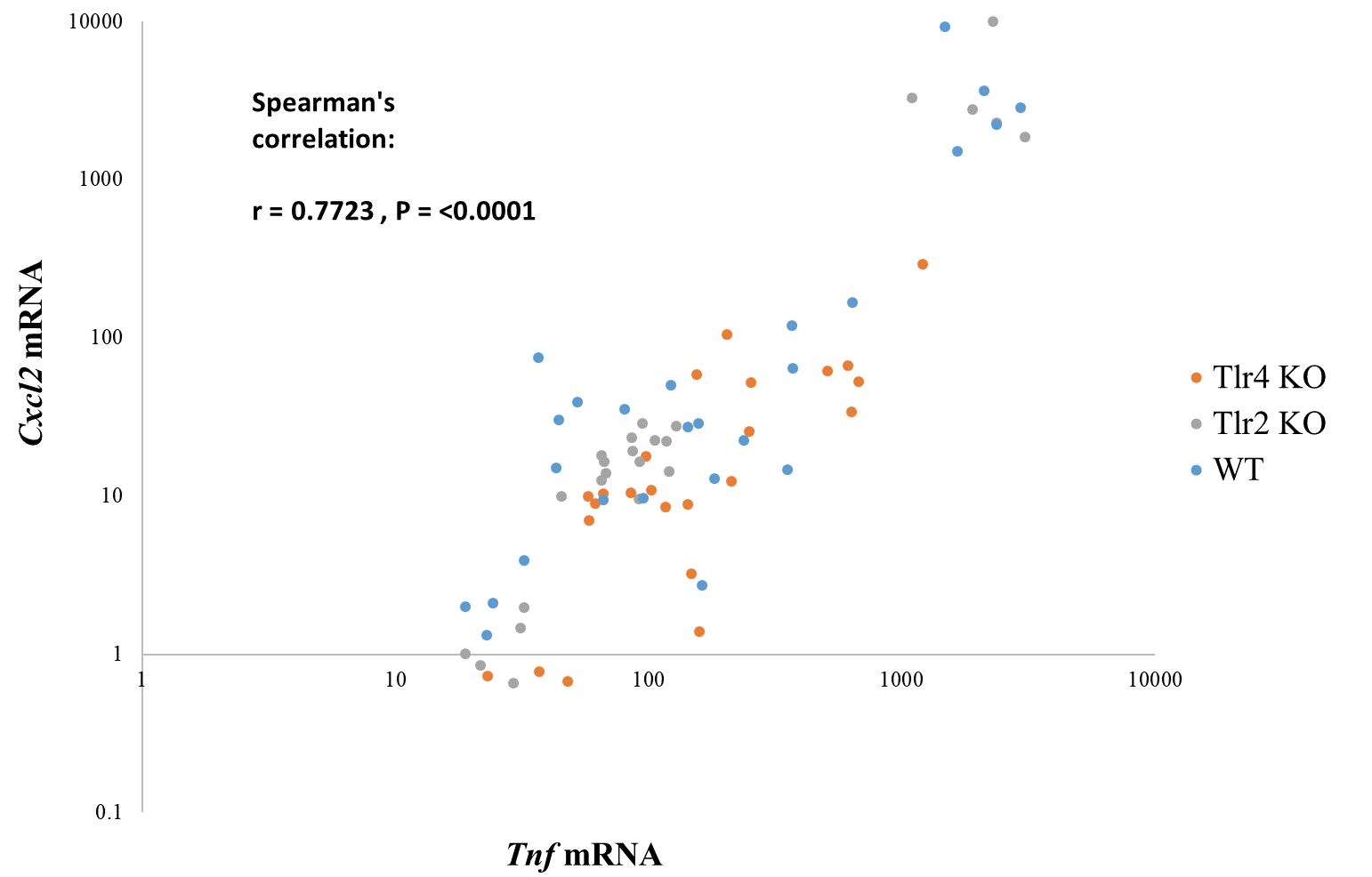

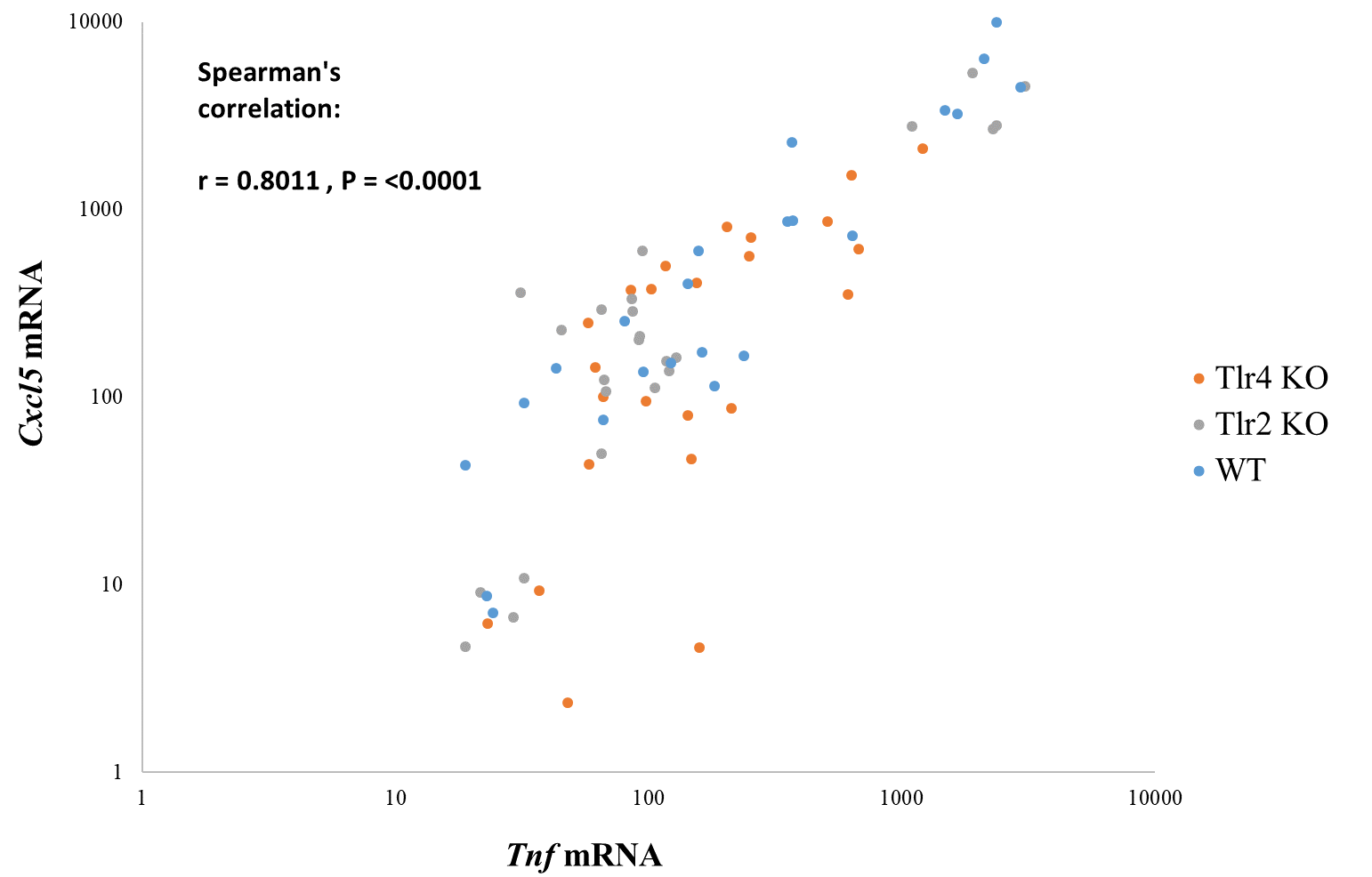


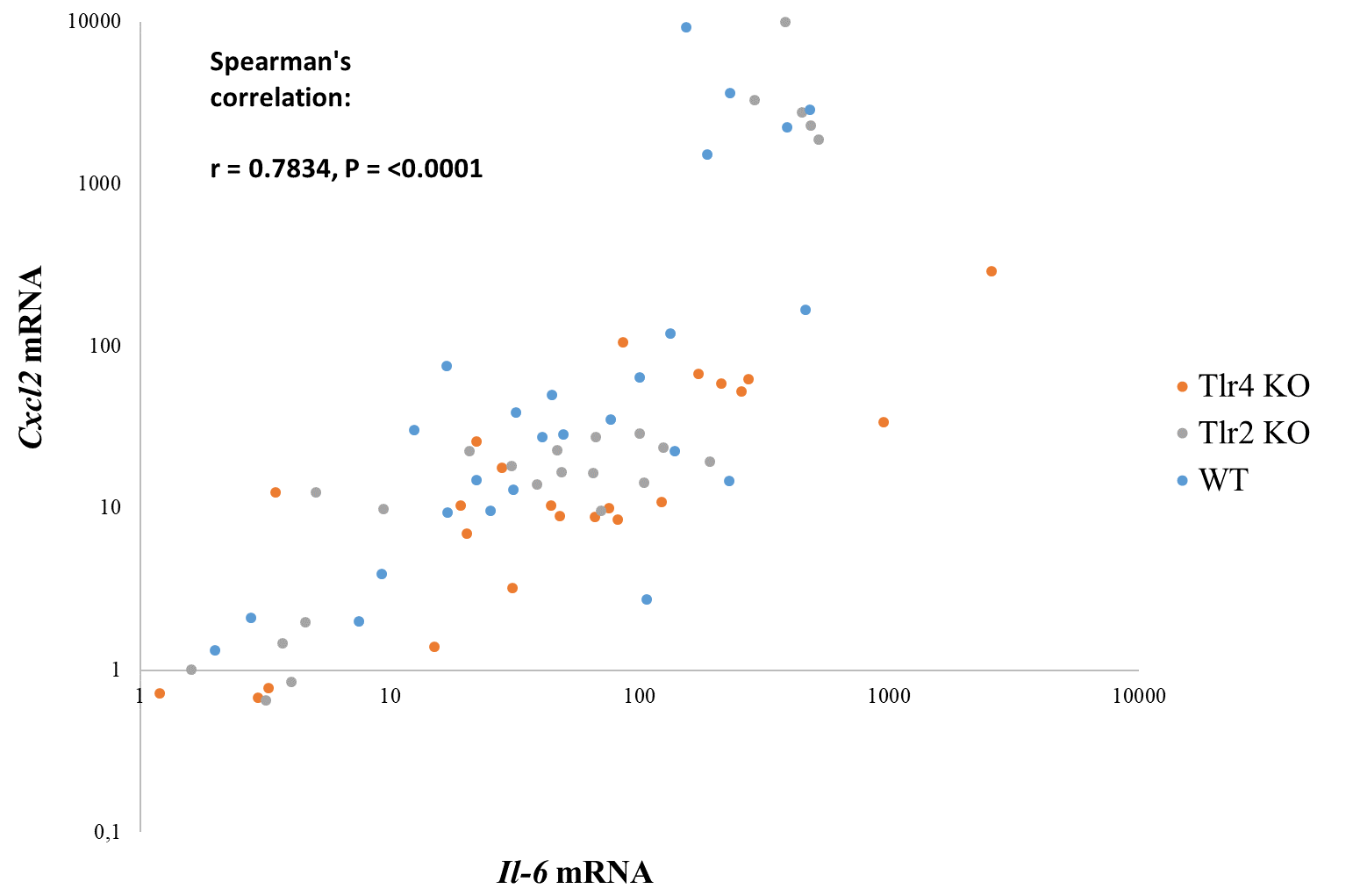

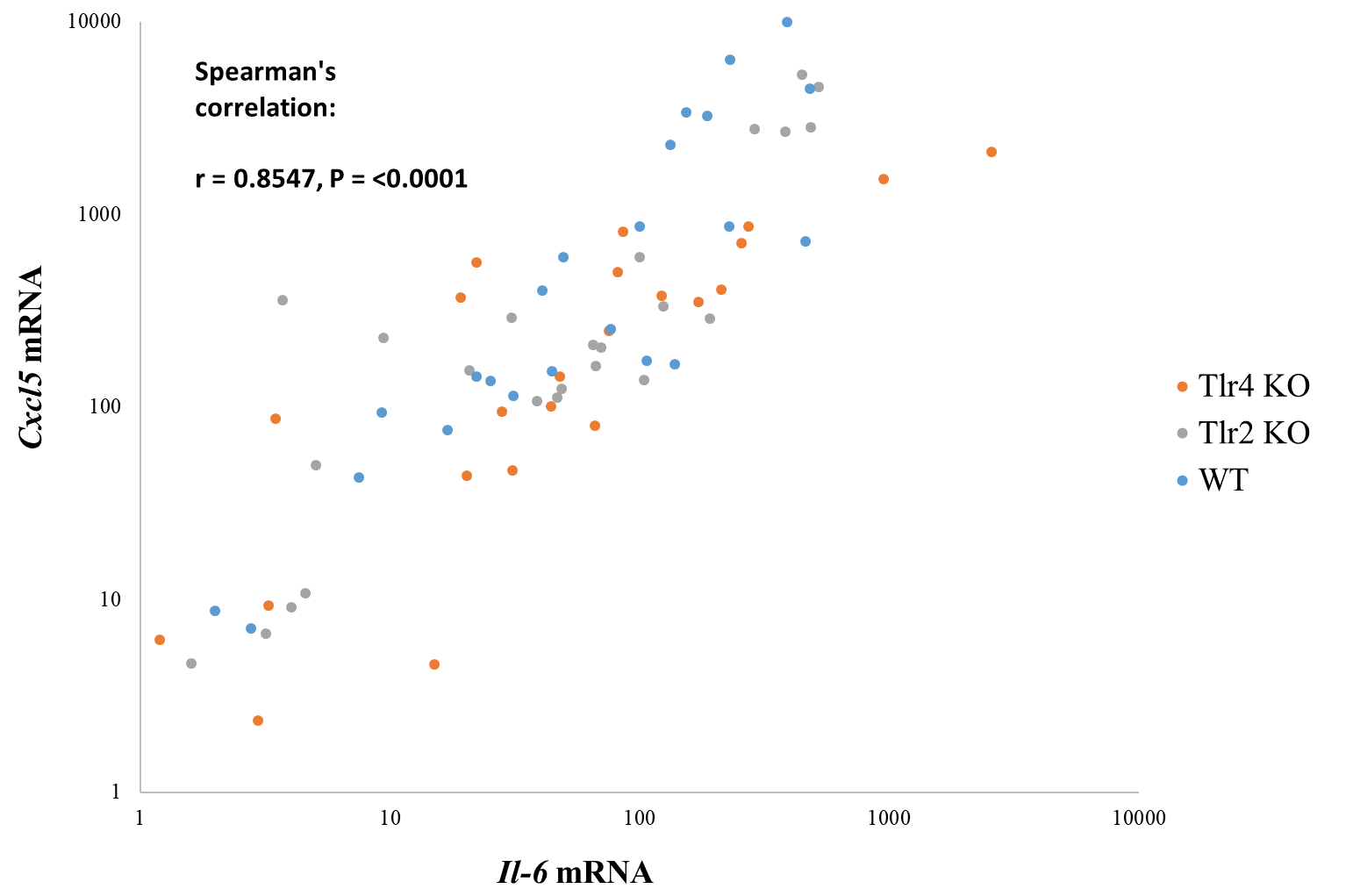


**Fig. S6** Correlations between *Cxcl2*, *Cxcl5*, *Il-6* and *Tnf* mRNA levels in lung tissue (double logarithmic scale).


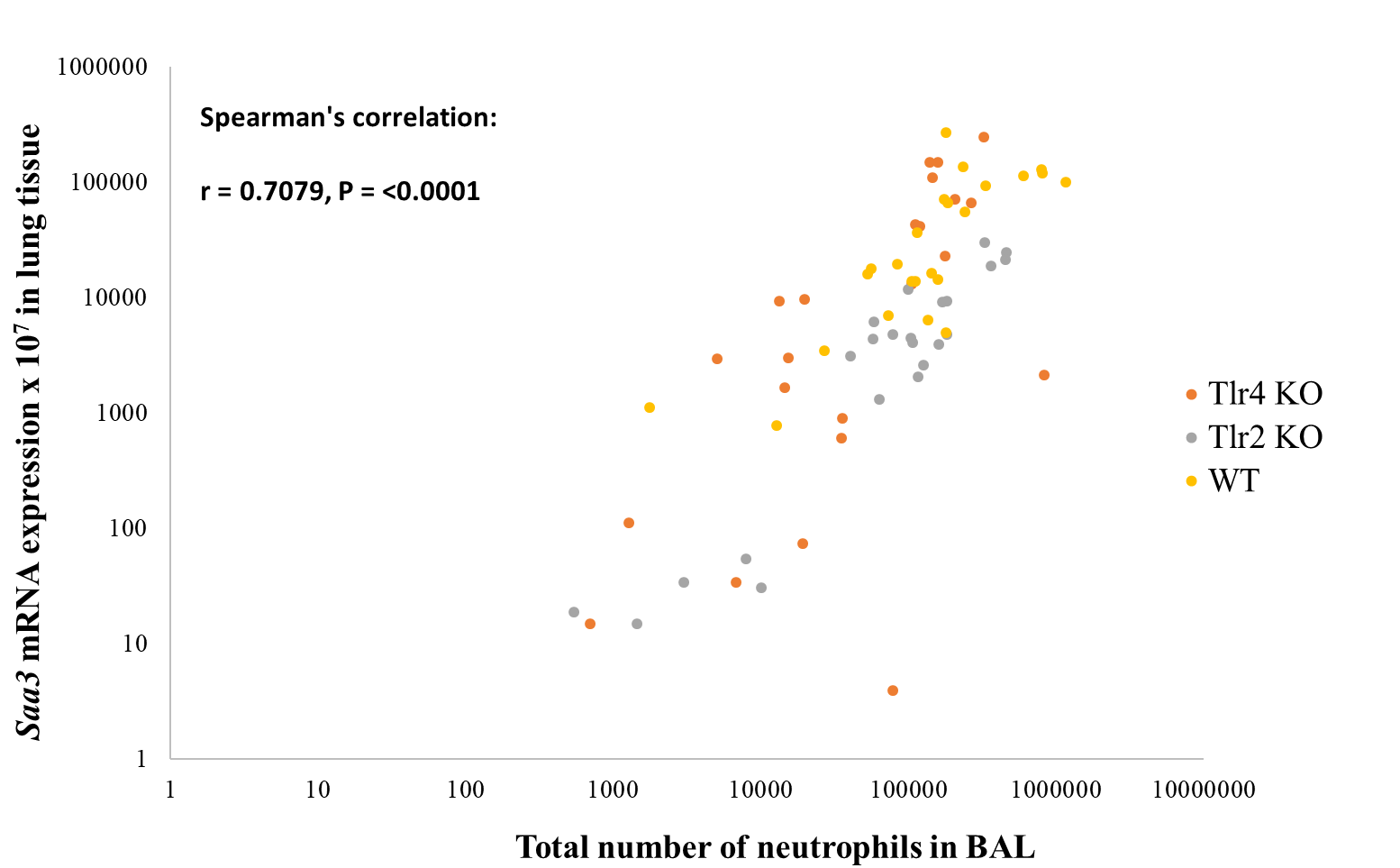

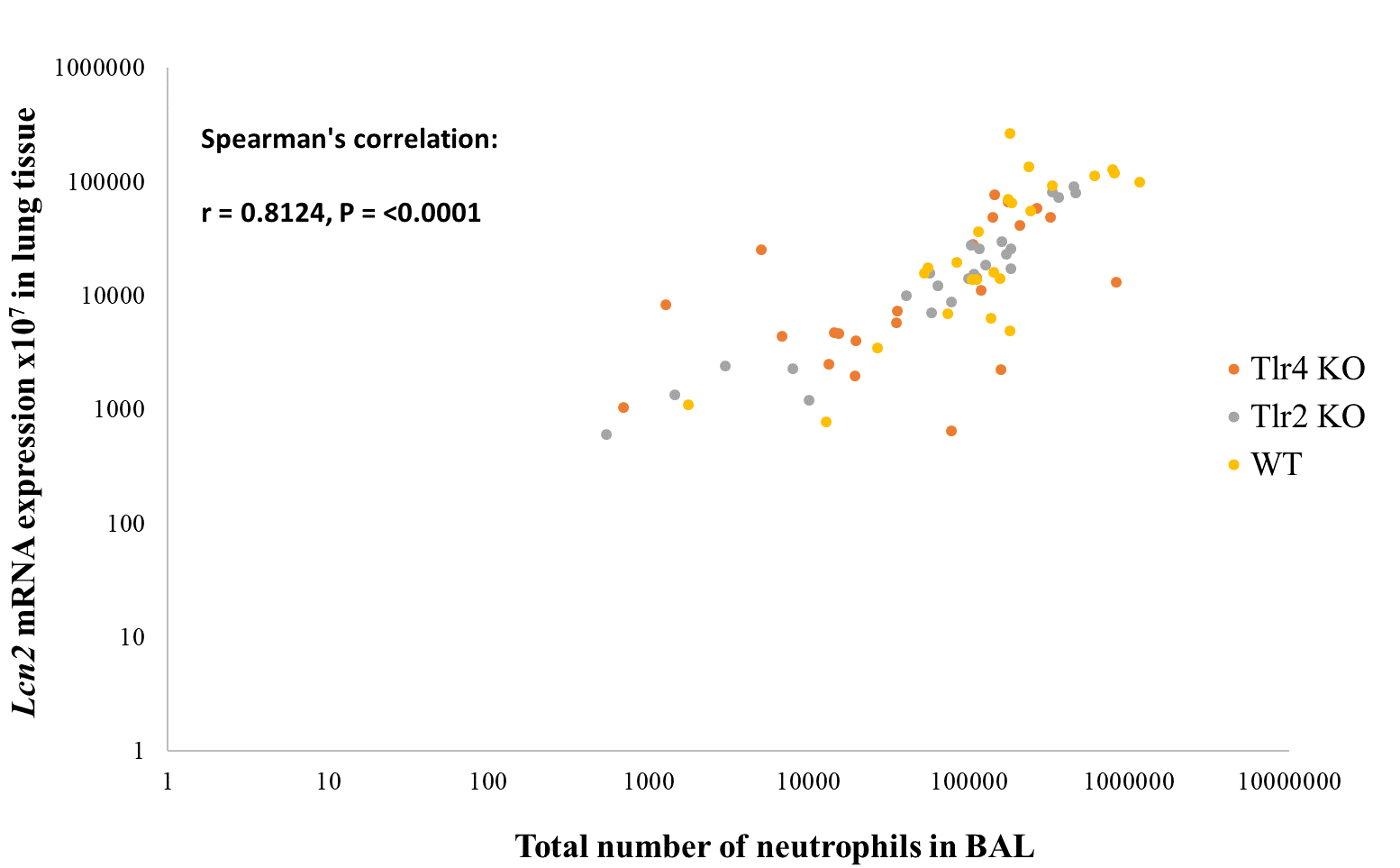
**
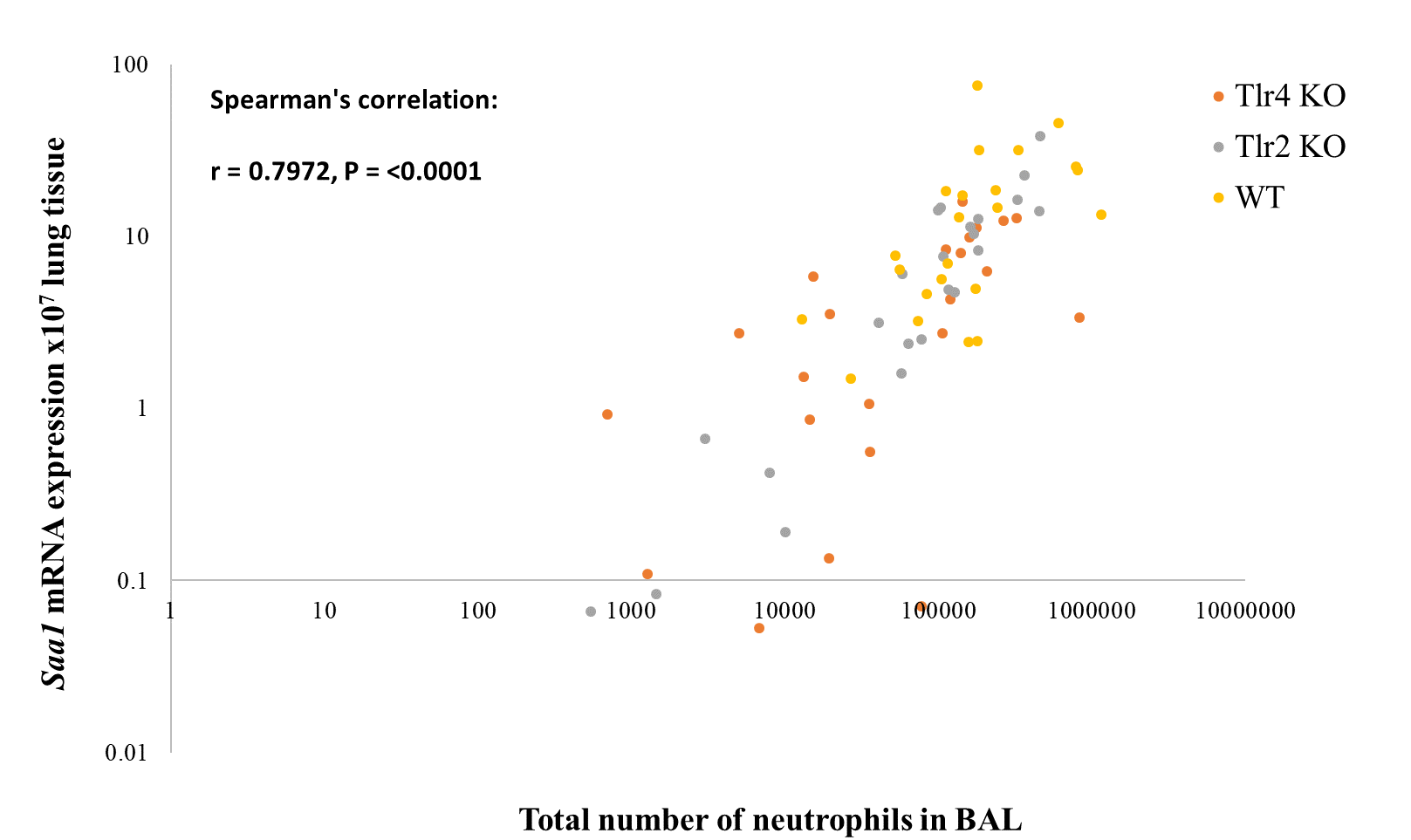
**

**Fig. S7** The correlation between influx of neutrophils and expression of acute phase response genes (*Saa3*, *Lcn2*, *Saa1* mRNA) in lung tissue (double logarithmic scale).


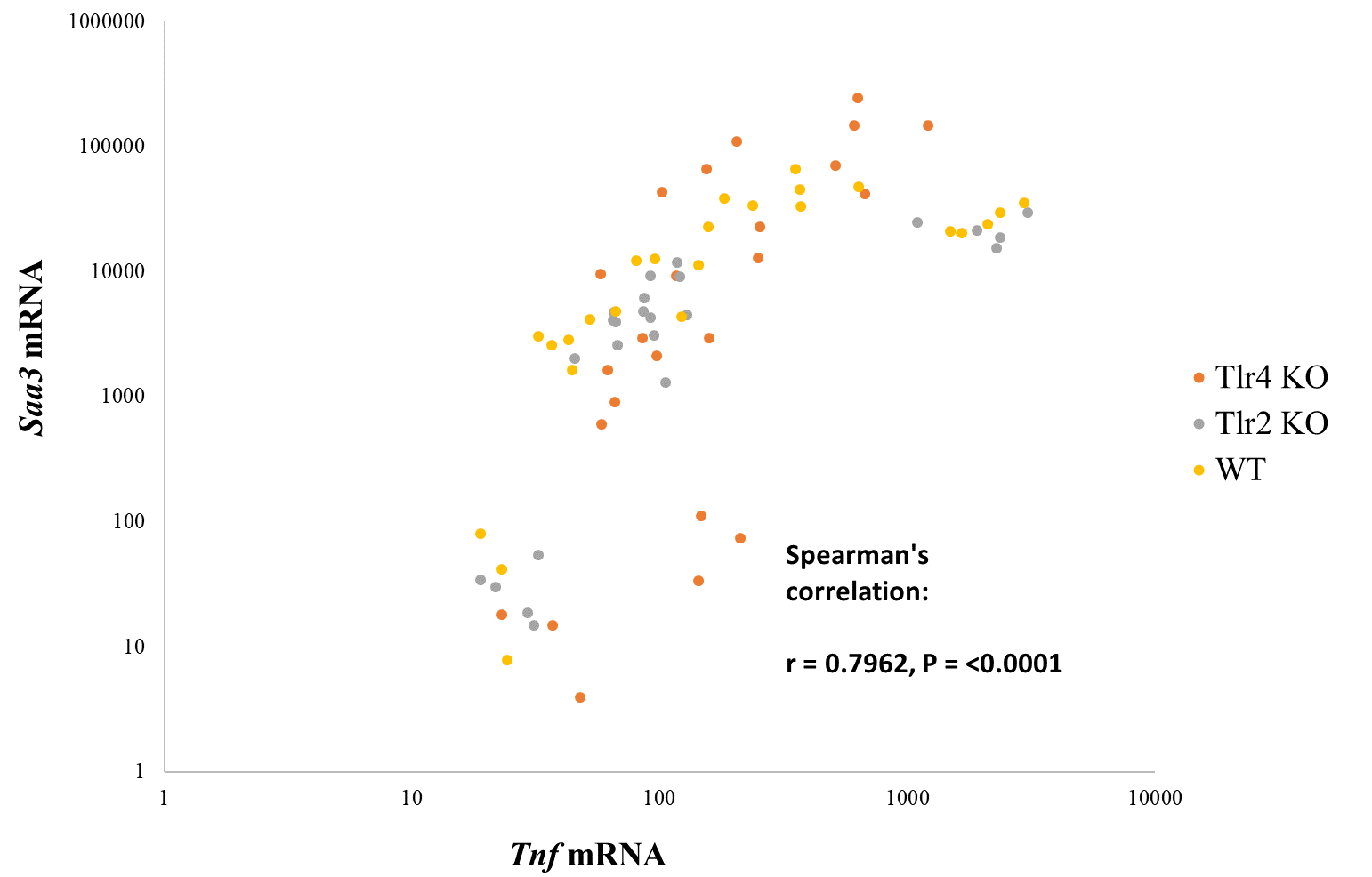

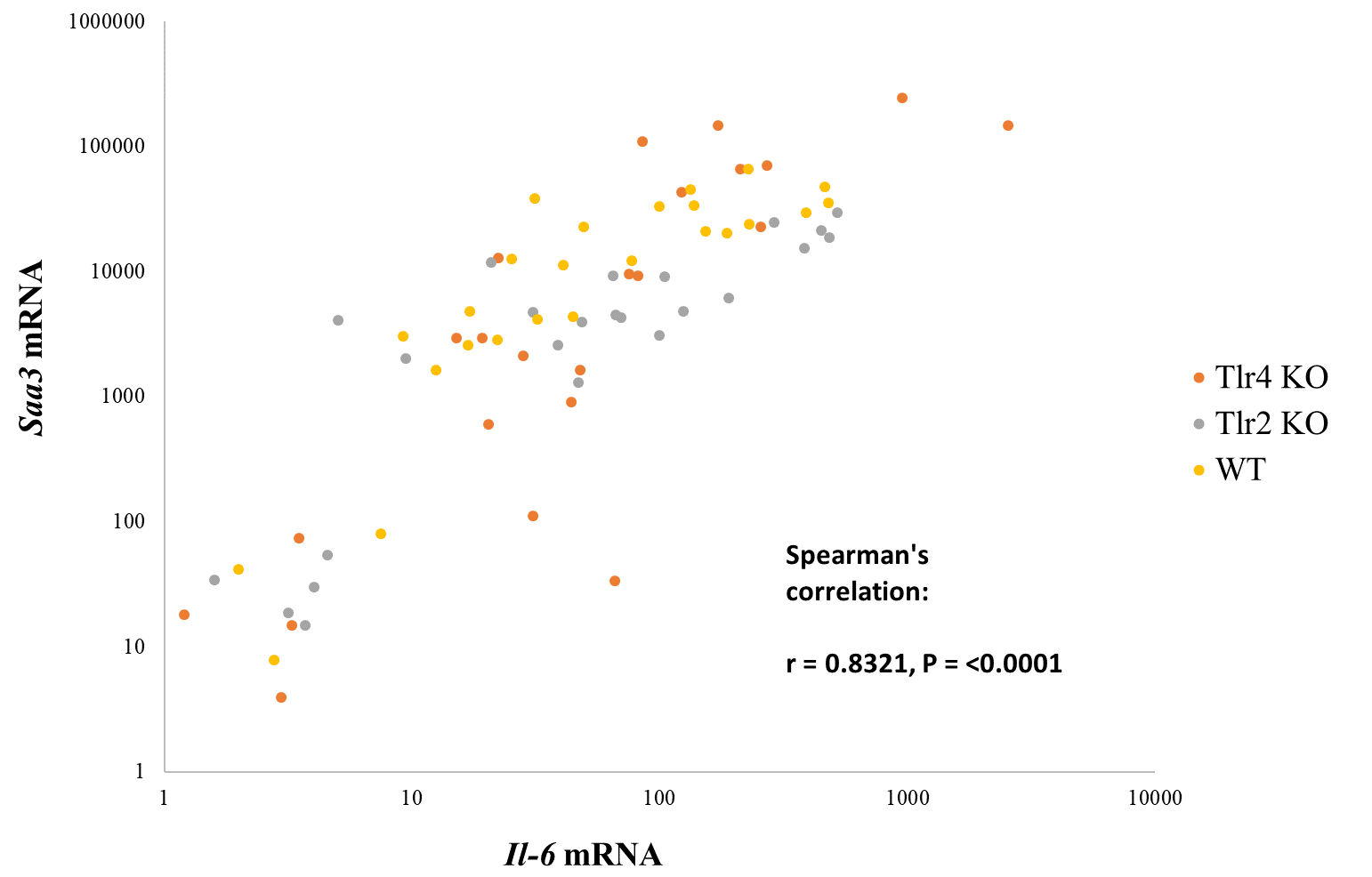


**Fig. S8** Correlations between *Tnf*, *Il-6* and *Saa3* mRNA in lung tissue, respectively (double logarithmic scale).


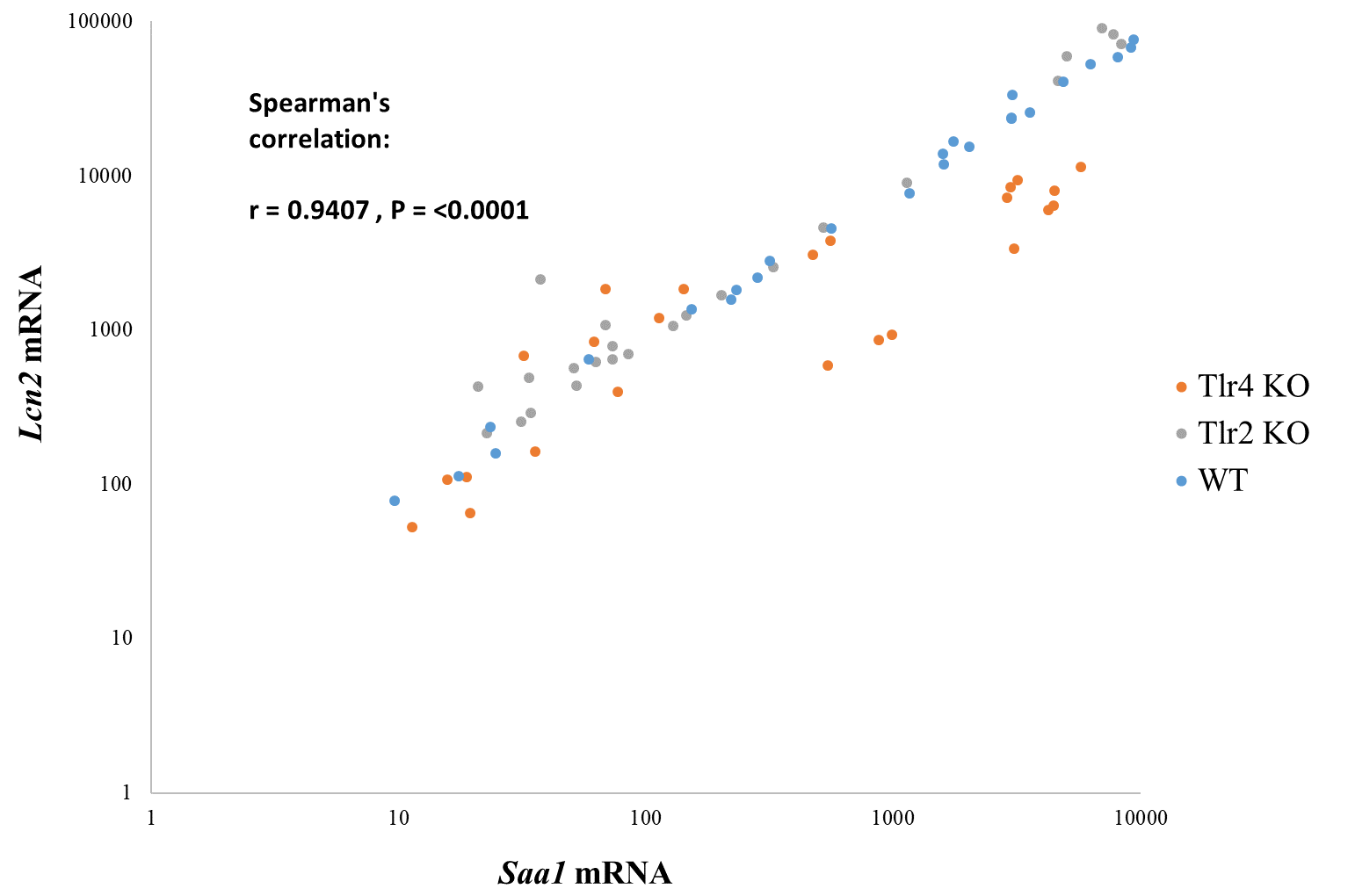


**Fig. S9** Correlations between *Saa1* and *Lcn2* mRNA expression in liver tissue (double logarithmic scale).
